# Dynamics of Dark-Fly Genome Under Environmental Selections

**DOI:** 10.1534/g3.115.023549

**Published:** 2015-12-04

**Authors:** Minako Izutsu, Atsushi Toyoda, Asao Fujiyama, Kiyokazu Agata, Naoyuki Fuse

**Affiliations:** * Department of Biophysics, Graduate School of Science, Kyoto University, Kyoto 606-8502, Japan; † Comparative Genomics Laboratory, National Institute of Genetics, Mishima 411-8540, Japan; ‡ Department of Genetics, Graduate University for Advanced Studies, Mishima 411-8540, Japan; § Department of Cell and Developmental Biology, Graduate School of Biostudies, Kyoto University, Kyoto 606-8501, Japan

**Keywords:** *Drosophila*, environmental adaptation, reselection experiment, genome-wide analysis, experimental evolution

## Abstract

Environmental adaptation is one of the most fundamental features of organisms. Modern genome science has identified some genes associated with adaptive traits of organisms, and has provided insights into environmental adaptation and evolution. However, how genes contribute to adaptive traits and how traits are selected under an environment in the course of evolution remain mostly unclear. To approach these issues, we utilize “Dark-fly”, a *Drosophila melanogaster* line maintained in constant dark conditions for more than 60 years. Our previous analysis identified 220,000 single nucleotide polymorphisms (SNPs) in the Dark-fly genome, but did not clarify which SNPs of Dark-fly are truly adaptive for living in the dark. We found here that Dark-fly dominated over the wild-type fly in a mixed population under dark conditions, and based on this domination we designed an experiment for genome reselection to identify adaptive genes of Dark-fly. For this experiment, large mixed populations of Dark-fly and the wild-type fly were maintained in light conditions or in dark conditions, and the frequencies of Dark-fly SNPs were compared between these populations across the whole genome. We thereby detected condition-dependent selections toward approximately 6% of the genome. In addition, we observed the time-course trajectory of SNP frequency in the mixed populations through generations 0, 22, and 49, which resulted in notable categorization of the selected SNPs into three types with different combinations of positive and negative selections. Our data provided a list of about 100 strong candidate genes associated with the adaptive traits of Dark-fly.

The adaptive responses of organisms are thought to be a driving force for evolution. The characteristic traits of organisms, such as physiology, morphology, and behavior, impact organisms’ fitness in any particular environment, and are selected during the process of evolution. To understand the mechanisms of environmental adaptation, researchers have generally examined the traits of organisms that have evolved in nature. One of the best-known examples is the shape of the beaks of Darwin’s finches, which are related to the feeding habitats of each species (Grant 2003; Lamichhaney *et al.* 2015). However, it is laborious to clarify the precise roles of a particular trait in environmental adaptation, because adaptation is ultimately the output resulting from multiple traits, and because the natural environment includes fluctuating features.

The genetic basis of environmental adaptation is even more complicated. Recent progress in next generation sequencing (NGS) technology enables us to examine the whole genome as a means of studying environmental adaptation. Genome-wide association studies (GWAS) have revealed genome differences between two populations without any *a priori* information about traits, and have linked genes with traits that vary in populations (Burton *et al.* 2007; McCarthy *et al.* 2008). For example, GWAS successfully identified genes associated with hypoxia tolerance in Tibetan people and thermal regulation in naked mole rat (Simonson *et al.* 2010; Kim *et al.* 2011). Quantitative trait loci (QTL) analysis is a traditional approach to identify genes that contribute to the evolved traits of organisms, for example, the genes involved in the pelvic loss of stickleback and in the albino skin of cave fish (Colosimo *et al.* 2004; Borowsky and Wilkens 2002). Although these approaches have provided great insights for studying environmental adaptation, it is still unclear how the combination of multiple genes contributes to the fitness in an environment and how the adaptive genes are selected during the course of evolution (Barton and Keightley 2002; Barrett and Hoekstra 2011).

Experimental evolution studies are a powerful approach to observe genome selection during evolutionary processes (Barrick *et al.* 2009; Tenaillon *et al.* 2012; Araya *et al.* 2010; Barrick and Lenski 2013). In this kind of study, organisms evolve in a defined environment, and the genome alterations and selections are analyzed across a time course. Early experimental evolution studies using *Drosophila* revealed several important concepts of evolutionary biology, such as genetic assimilation (Waddington 1953) and evolutionary capacitors (Rutherford and Lindquist 1998), and now experimental evolution studies are achieving great progress by means of genome analysis technology. Although some studies have characterized the relationship between genes and evolved traits (Burke *et al.* 2010; Zhou *et al.* 2011; Orozco-Terwengel *et al.* 2012), genome research on experimentally evolved sexual organisms is still limited.

A *Drosophila melanogaster* line has been maintained in constant dark conditions for more than 60 years (1500 generations), since 1954, by a laboratory at Kyoto University (Mori 1986; Fuse *et al.* 2014). We designated this fly line “Dark-fly”, and utilize it to investigate molecular mechanisms underlying environmental adaptation. Dark-fly is an invaluable material of a long-term experiment, and the term is even longer than that of a similar historic study performed by Fernandus Payne (Payne 1911). Although Dark-fly has no apparent morphological features related to dark-adaptation, previous studies revealed that Dark-fly possesses strong phototactic behavior and somehow retains circadian locomotor rhythm (Mori 1983; Imafuku and Haramura 2011). We also reported high fecundity of Dark-fly in constant dark conditions (Izutsu *et al.* 2012), implying that Dark-fly possesses some traits advantageous for living in the dark. To explore the genetic basis of Dark-fly’s traits, we previously performed whole-genome sequencing, and identified approximately 220,000 single nucleotide polymorphisms (SNPs) and 4000 insertions or deletions (InDels) in the Dark-fly genome (Izutsu *et al.* 2012). However, at that time we did not identify which SNPs and InDels contribute to environmental adaptation of Dark-fly. A serious problem of the long-term Dark-fly project in this regard is that the control sister flies were accidentally lost over the course of the 60 years of maintaining the Dark-fly line (Fuse *et al.* 2014). Therefore, it is impossible to precisely examine the genome evolution in the Dark-fly history and to accurately compare genomes and traits between Dark-fly and its control sisters.

To circumvent this problem, we designed an experiment to accomplish reselection of the dark-adapted genome. That is, we reared mixed populations of Dark-fly and a wild-type fly in constant dark (DD) and normal light–dark cycling (LD) conditions. We compared the population genome using an analogy of QTL analysis, and detected condition-dependent selection of the fly genome during the time course. The results obtained demonstrated the usefulness of genome reselection experiments for studying environmental adaptation, and provided us with a list of potential candidate genes involved in Dark-fly’s adaptive traits.

## Materials and Methods

### Flies

*D. melanogaster* Dark-fly Oregon-R-S line (Dark-fly) has been reared in constant dark conditions at 25° with a minimal nutrient medium, Pearl’s medium (Pearl 1926) (termed M condition hereafter), since 1954 (Mori and Yanagishima 1959). We have also reared Dark-fly in constant dark conditions with a standard cornmeal medium (termed F condition) since 2008 (Izutsu *et al.* 2012). Just before starting the experiments reported here, Dark-fly was reared under 12-hour light–dark cycling conditions with standard cornmeal medium (LD condition) for 3–18 generations to examine the genetically fixed traits. We designate the Dark-fly that was reared in the M condition for 1351 generations, in the F condition for 56 generations, and in the LD condition for three generations as “D1351+56-3”. D1351+56-3 was used for the fitness assay against Oregon-R-S. The Dark-fly used for the fitness assay against Urbana-S was D1351+81-18 (so designated using the same nomenclature system), and that used for the mixed population experiment was D1388-3.

The Oregon-R-S, Canton-S-iso3, and Urbana-S strains used for the experiments were obtained from the Bloomington Stock Center (stocks #4269, #6366, and #4272). The transgenic fly lines expressing Green Fluorescent Protein (GFP) and *Discosoma* red fluorescent protein (DsRed) [P(GFP)Zasp66(ZCL0663) and P(DsRed)ap(PyR10)] were obtained from the Kyoto Stock Center (stocks #110740 and #109136). To make competitor lines for the fitness assay, both transgenic lines were backcrossed with the Oregon-R-S or Urbana-S line more than 10 times. We called these flies GFP-Oregon-R-S, DsRed-Oregon-R-S, GFP-Urbana-S, and DsRed-Urbana-S.

### Fitness assay

For the fitness assay, we put four kinds of flies (five individuals each of females carrying GFP, males carrying DsRed, tester females, and tester males) into the same culture vial (about 30 ml volume), and kept them in DD or in 12-hour LD conditions for 3 d. Then, the parental flies were discarded and the number of progeny was counted after the adult offspring emerged. We observed the adult offspring under a fluorescence microscope (Olympus SZX16) and classified them according to their expression of GFP and DsRed (see *Results*). We evaluated the fitness of tester parents based on the number of these classified progeny. Statistical tests for the proportions of offspring were performed using the Mann–Whitney *U*-test (wilcox.exact function of the R program: http://www.r-project.org/) and the Welch *t*-test (t.test function with var.equal = F option). We also calculated the comparative fitness (CF) of the parental tester strain in the offspring population using the following equation:

CF={2×W+1×(G+R)+0×Y}/{2×(W+G+R+Y)}×100

where *W* is the number of White progeny (from tester females and tester males), *G* is the number of Green progeny (from GFP females and tester males), *R* is the number of Red progeny (from tester females and DsRed males), and *Y* is the number of Yellow progeny (from GFP females and DsRed males). Thus, CF = 50% means that the tester and competitor flies had equal fitness.

For the assay against competitor with Urbana-S background, we used a modified fly food (46 g corn flour, 23 g corn grits, 40 g dry yeast, 100 g D-glucose, 7.2 g agar, 0.5 g butyl benzoate, 5 ml propionic acid / 1 L final volume in water).

Canton-S-iso3 was used as a tester strain to examine the effects of distinct genetic backgrounds between tester and competitor strains.

### Mixed population experiment

First, we prepared 16 vials each containing a total of 20 flies (five female and five male hybrids between Oregon-R-S female and Dark-fly male, plus five female and five male hybrids between Dark-fly female and Oregon-R-S male) as one replicate population. Three such replicate populations each were placed in LD and DD conditions. The parental flies were removed from the vials after 3 d and stored at −80° for use in the subsequent analyses. After the adult progeny emerged, we collected all flies from the 16 vials together into one vial, mixed them carefully, and divided them again into 16 fresh vials every generation with ice anesthesia, so that the flies reared in the 16 vials were kept as one population. One vial usually contained about 60 offspring flies, and thus one replicate population in 16 vials contained about 1000 flies. The mixed populations were maintained by transfer to fresh vials every 11–16 d. After generation 50, we started to use the modified fly food described above.

To estimate the size of mixed populations, we measured the weight of frozen flies. Each value of weight was converted to number of flies using the mean weight of six populations at generation 0 (320 flies = 0.42 g). The statistical test of population size was performed using the one-way ANOVA of Microsoft Excel.

### Genome sequencing for mixed populations

To extract genomic DNA from the mixed populations, about 1000 frozen flies were crushed with liquid nitrogen and the fly powder was thoroughly mixed. The genomic DNA was extracted in quadruplicate from four small portions of the fly powder according to our previously reported standard method (Izutsu *et al.* 2012) and the DNA from these four extracts were combined. Paired-end sequencing libraries were constructed according to Illumina’s standard protocols. Sequencing was performed using the Illumina HiSequation 2000 system. Raw sequence data were deposited in DNA Data Bank of Japan under accession number DRA004032 (DRR046855-DRR046867).

Raw data of read sequences were aligned on the reference genome (Flybase FB2009_09 October, Dmel Release 5.22) using the aln and sampe functions (with default parameters) of BWA software (bwa-0.5.9rcl) (Li and Durbin 2009), and the files obtained were converted to the variant sequence data (pileup or mpileup files) using samtools-0.1.16 (Li *et al.* 2009). To calculate SNP frequency, we used the pileup function (with –B options) of samtools and the pileup2snp (minimum allele count ≥ 0, minimum coverage ≥ 5, minimum frequency ≥ 0) function of VarScan (ver. 2.3.5) (Koboldt *et al.* 2009). To extract Dark-fly-specific SNPs, we used the compare functions of VarScan with a slight modification for comparing the specific allele.

### SNP analyses

Nonmetric multidimensional scaling (MDS) analysis was performed using the isoMDS function in the MASS package of R. Other analyses of overall SNP frequency were performed using standard functions of the R program.

For Fisher’s exact test of SNP frequency, we first merged bam files of replicate populations with the merge function and mpileup function (with –B –d 9000) of samtools-0.1.19, and performed Fisher’s exact test (max-coverage 9000) using popoolation2 software (Kofler *et al.* 2011). We used p-values to identify SNPs showing a significant difference in frequency (Bonferroni-corrected p-value < 0.01, and top 5% in ranking) between LD- and DD-reared populations. Quantile–quantile (QQ) plotting of p-values was done using an R script (Saxena *et al.* 2007). The chi-square test was performed using chisq.test function of the R program. We grouped SNPs showing the top 5% p-values of Fisher’s exact test into three types: type 1, 2, and 3 (0.8–1.0, 0.35–0.8, and 0–0.35 frequency in DD-reared populations, respectively).

### Identifying candidate regions

We assumed that two significant SNPs that were located within 100 kb of each other were linked, and grouped them into the same region. We then listed the candidate regions that possibly included dark-adaptive genes. We also compared these candidate regions to the previously identified runs of homozygosity (ROH) regions (Izutsu *et al.* 2012). Effects of SNPs and InDels were examined using snpEff software (Cingolani 2012).

To narrow down the candidate regions, we used the MULTIPOOL program, ver. 0.10.2 (Edwards and Gifford 2012), which estimates a QTL locus from the allele count data using a dynamic Bayesian network model. This program is known to be sensitive for fixed SNPs (Albert *et al.* 2014), and therefore we filtered the data with SNP frequency < 0.01 or > 0.99 before performing the analyses. We set the parameters of the program as -n 3000 -r 1000 -c 12000 -m contrast, because we processed the data of about 3000 flies and roughly estimated the recombination rate after 49 consecutive generations (Hey and Kliman 2002). We initially obtained logarithm of odds (LOD) scores of 1-kb windows for each chromosome arm. After detection of the LOD score peaks (LOD score > 75 and interval > 1 Mb), we recalculated the LOD scores from the data of 1 Mb regions centered at the peak, and obtained the 90% credible interval spans for each peak, except for peak one. For analysis of peak one, we used the data of a 3-Mb region, because high LOD scores in this region were extended longer than that in other peaks.

### qPCR-based measurements of SNP frequency

To confirm the reliability of SNP frequency calculated from Illumina NGS data, we also measured SNP frequency by the quantitative PCR (qPCR)-based method (Germer *et al.* 2000). We analyzed the SNP frequency of 116 samples in total, consisting of nine SNPs each of 12 or 13 population genomes. To detect a SNP, we used two pairs of primers: one consisting of the reference sequence and another one carrying the Dark-fly SNP at its 3′ end, each paired with a common reverse primer. Although the difference between the primers was only one nucleotide, PCR amplification was affected by whether the 3′ end of the primer matched or not. From the difference in the number of amplification cycles between these two PCR reactions, we could estimate the SNP frequency of the template DNA. Before examining the population genome, we tested the usefulness of primer pairs using genomic DNAs of Dark-fly and Oregon-R-S (Dark-fly SNP frequency: 1.0 and 0.0, respectively). If the difference in the number PCR amplification cycles was large enough (more than 4.25 cycles), the primer pairs were used for the analysis. The primers used are listed in Supporting Information, Table S1. Each PCR reaction mixture (total 10 μl) consisted of genomic DNA (1 ng/μl) and primers (0.3 μM each) in 1 × QuantiTect SYBR Green PCR master mix (Qiagen), except for the SNP at 2R:12478534. For this SNP, the PCR reaction mixture consisted of genomic DNA (1 ng/μl), primers (0.3 μM each), dNTP Mix (0.2 mM), Rox dye (Toyobo), EvaGreen dye (Biotium), and DNA polymerase Stoffel Fragment (Applied Biosystems) (1 unit/μl) in the Stoffel Buffer with 2.5 mM MgCl_2_. qPCR was performed using an ABI 7900HT. The data were normalized by the value of a hybrid line between Dark-fly and Oregon-R-S (Dark-fly SNP frequency: 0.5).

### Data availability

Raw sequence data are available at http://trace.ddbj.nig.ac.jp/DRASearch/.

## Results

### Fitness of Dark-fly under dark environment

**Figure 1:**
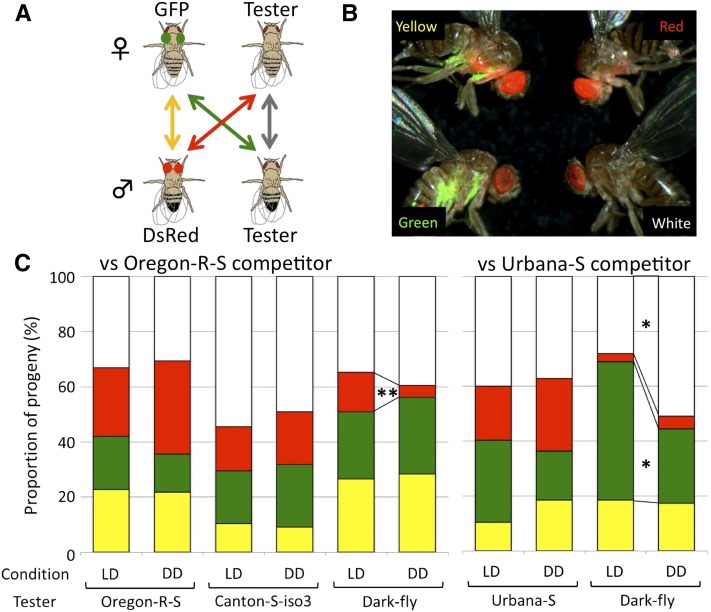
Competition assay for measuring relative fitness. (A) Four kinds of flies (competitor females carrying GFP, competitor males carrying DsRed, tester females, and tester males) were reared together and mating could occur in any combination of them. From the numbers of progeny, the relative fitness of tester parents was calculated. (B) An example of observations of progeny. The views under bright-field and fluorescent lights were merged in the image. According to the fluorescent markers, progeny were categorized into four groups. (C) The mean proportion of progeny in each test. Left: assays (n = 10) against the Oregon-R-S competitors (GFP-Oregon-R-S females and DsRed-Oregon-R-S males). Right: assays (n = 5) against the Urbana-S competitors (GFP-Urbana-S females and DsRed-Urbana-S males). The colors correspond to the combinations of parental flies shown in (A) and the terms used to designate the progeny group (Yellow, Green, Red, and White). *p-value < 0.05; **p-value < 0.01, Mann–Whitney *U*-test.

To examine whether Dark-fly has high fitness in dark conditions, we developed an assay system to measure the relative fitness under mating competition. For this assay, we utilized transgenic fly lines expressing fluorescent proteins, GFP and DsRed, as competitor lines, and put them together with tester flies into the same culture vial (competitor females carrying GFP, competitor males carrying DsRed, tester females, and tester males; Figure 1A). These parental flies could mate freely in any combination, and therefore the relative fitness of the tester compared to the competitor could be estimated from the relative proportion of offspring. After adult offspring emerged, they were classified according to their expression of GFP and DsRed (Figure 1B). Briefly, the flies expressing only GFP (termed “Green progeny” hereafter) would be from parents of a GFP female and a tester male, the flies expressing only DsRed (termed “Red progeny”) would be from a tester female and a DsRed male, the flies expressing both markers (termed “Yellow progeny”) would be from a GFP female and a DsRed male, and the flies expressing neither marker (termed “White progeny”) would be from a tester female and a tester male. We performed the competition assay under different light conditions: DD or normal LD conditions, and examined the condition-dependent shift of the proportion of each type of offspring.

We prepared two pairs of competitor strains by backcrossing with wild-type strains, Oregon-R-S or Urbana-S (see *Materials and Methods*), and then tested five combinations of competitor and tester (Figure 1C). Each combination produced a particular number and proportion of offspring (Figure S1 and Table S2). Even when the same strains were used as competitor and tester (for example, Oregon-R-S competitor *vs.* Oregon-R-S tester), the proportions of Yellow/Green/Red/White offspring were not equal (Figure 1C). These unequal proportions were probably due to the remaining genetic background of competitor lines and/or the effects of the GFP and DsRed transgenes. Importantly, we did not find any significant difference in these proportions between the LD and DD conditions in most cases. The exceptions were the cases of the Dark-fly tester. In the assay for Dark-fly tester and Oregon-R-S competitor, the proportion of the progeny changed according to the light conditions (Figure 1C). The proportion of Red progeny was significantly decreased in the DD compared to the LD condition (Mann-Whitney *U*-test, p-value = 0.0062, n = 10, Table S3). Conversely, the proportions of the White and Green progenies tended to be increased, though the differences were not statistically significant. These alterations of offspring proportion might have been due to different reproductive ability of Dark-fly in different light conditions (see *Discussion*). When we examined Dark-fly tester and Urbana-S competitor, the proportion of White progeny was significantly increased in the DD condition compared to the LD condition (p-value = 0.0159, n = 5, Table S3 and Figure 1C), whereas the proportion of the Green progeny was decreased instead (p-value = 0.0318). These statistical evaluations were reconfirmed by the Welch *t*-test after the angular transformation of proportions (data not shown). These results indicate that regardless of which competitor line was used, Dark-fly exhibited a unique difference of its ability to reproduce in LD *vs.* DD conditions.

From the above data, we also calculated the CF of tester *vs.* competitor (CF of 50% indicates equal fitness of tester and competitor; see *Materials and Methods*). The CF of Dark-fly *vs.* Oregon-R-S competitor showed a slight increase, from 54.1% in the LD to 55.6% in the DD condition (Table S2). Against Urbana-S competitor, the CF of Dark-fly was significantly increased, from 54.8% in the LD condition to 66.8% in DD (Mann–Whitney *U*-test, p-value = 0.0318, Table S2). We did not detect such a condition-dependent advantage for other testers we examined. These results suggest that Dark-fly produces more progeny in the DD than in the LD condition and as a result dominates over the wild-type fly in the dark, and support the idea that Dark-fly is adapted to a dark environment.

### Mixed population experiment for identifying adaptive genes

**Figure 2:**
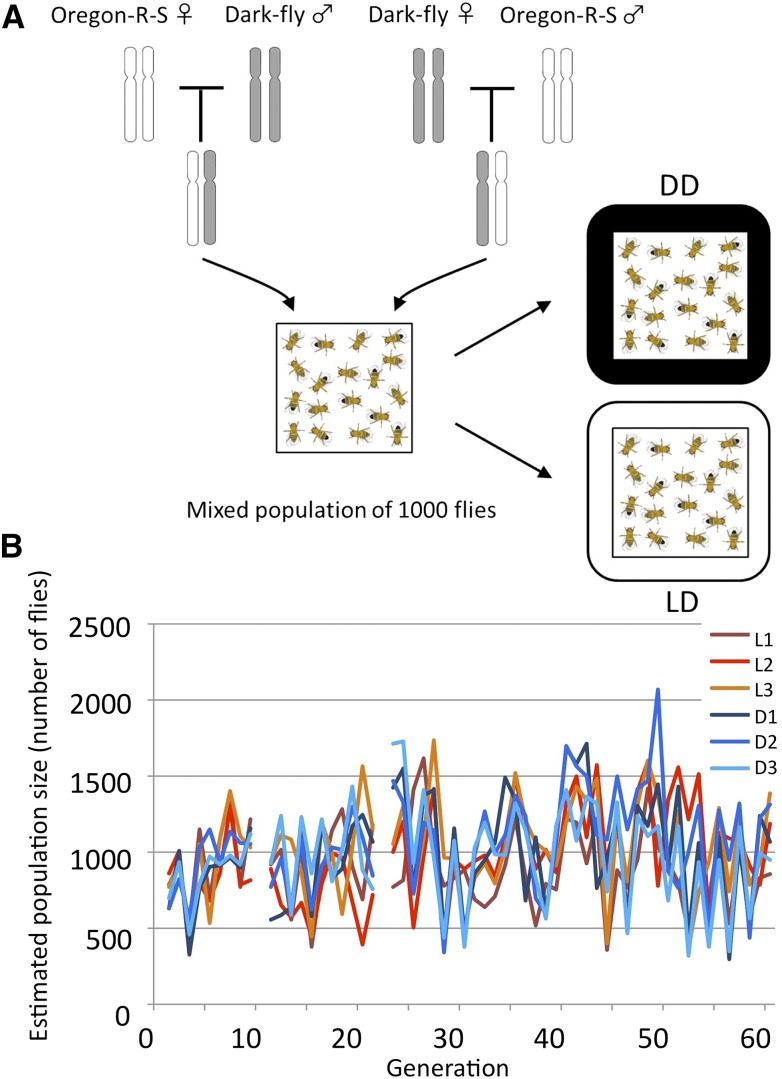
Mixed population experiment. (A) A schematic drawing of the mixed population experiment. The experiment started from hybrids of Dark-fly and Oregon-R-S. Those mixed populations were reared in LD and DD conditions during consecutive generations. (B) The history of the mixed population. The sizes of the mixed populations were estimated by measuring the weight of flies at every generation, except for generations 10 and 22. Three replicate populations reared in the LD condition are shown by dark-red, red, and light-red lines, and those reared in the DD condition are shown by dark-blue, blue, and light-blue lines, respectively.

To identify genes associated with Dark-fly’s adaptive traits, we designed a genome reselection experiment under differing environments. That is, we maintained large mixed populations of Dark-fly and Oregon-R-S (1:1 mixture starting from hybrid flies, Figure 2A) under different light conditions: LD and DD. During successive generations, the Dark-fly genome and Oregon-R-S genome would be mixed in the population, and selection would act on each genomic locus. We hypothesized that the genes involved in dark-adaptation would dominate in the DD-reared population, but not in the LD-reared population. To test this, we reared three replicate populations each in the LD and DD conditions, and stored (frozen) adult flies at every generation.

We examined the transition of population size during the first 60 generations by measuring the weight of the fly population (Figure 2B). The estimated average number of flies in a replicate population was 985, and the minimum and maximum numbers were 296 and 2069, respectively. There was no significant size difference among all populations (one-way ANOVA, p-value = 0.171, Table S4). Thus, the mixed populations were kept without showing any effect of the LD or DD conditions on population size.

We extracted the genomic DNA of the mixed population at generations 0, 22, and 49 (one replicate at generation 0, three replicates each in LD and DD conditions at generations 22 and 49, 13 populations in total) and performed whole-genome sequencing to measure the frequency of SNPs in each population’s genome. The average sequence coverage for the genome was 179 × in 13 populations (Table 1), so that we could detect a difference of SNP frequency for one out of 179 reads (0.006 frequency). First, we analyzed the SNP frequency at generation 0. All of the flies at generation 0 were hybrids of Dark-fly and Oregon-R-S, so we expected that Dark-fly SNPs would show a frequency of around 0.5 in the population. Among 217,329 SNPs previously identified in the Dark-fly genome (Izutsu *et al.* 2012), we found 143,685 SNPs (66.11%) that had a frequency of between 0.4 and 0.6 in the population at generation 0 (Figure S2). The remaining 73,644 SNPs did not show a frequency of around 0.5, and most of them showed a frequency of over 0.9, implying that these SNPs would be common between Dark-fly and Oregon-R-S. In previous analyses, we might have overlooked some SNPs, probably due to low depth of coverage (mean depth: 13.7). Therefore, we took the 143,685 SNPs with a frequency of 0.4 to 0.6 at generation 0 as Dark-fly SNPs (Figure S2, the black-outlined area) and used them in the following analyses.

**Table 1:** Summary of genome sequencing of the mixed population

Generation	Condition	Line	Read Length	Read Number	Mapped Read Number	Mapped Reads (%)	Mean Depth
0	—	—	100	386775696	361193875	93.39	214
22	LD	1	100	266855274	254924548	95.53	151
	LD	2	100	283632172	272484316	96.07	161
	LD	3	100	269666516	259954602	96.40	154
	DD	1	100	271880364	258818394	95.20	153
	DD	2	100	265563806	254379699	95.79	151
	DD	3	100	274437396	262753186	95.74	156
49	LD	1	100	351537438	329248183	93.66	195
	LD	2	100	386629584	362194949	93.68	215
	LD	3	100	269858658	243812205	90.35	144
	DD	1	100	418334430	386895826	92.48	229
	DD	2	100	345057530	317678449	92.07	188
	DD	3	100	379634634	354977577	93.51	210
Mean of total	—	—	—	320758731	301485831	94.14	179

Populations are indicated by generation number, condition (LD or DD condition), and replicate ID number. Read length and read number obtained from NGS data are shown for each population. Reads were mapped on the Flybase Dmel 5.22 genome (168,736,537 bases), and basic data of the mapping are shown. Mean depth of all data was 179.

We then calculated the frequency of each Dark-fly SNP in the replicate populations at generations 22 and 49. To evaluate the reliability of these frequencies calculated from the NGS data, we measured the frequency of some SNPs by a qPCR-based method (Germer *et al.* 2000). Briefly, we used two pairs of primers: one containing the reference sequence and the other carrying the Dark-fly SNP at its 3′ end, and estimated the SNP frequency in the population from the difference of the amplification cycles of qPCR using these two primer pairs. We found that SNP frequency measured by qPCR (total 116 samples) correlated well with the frequency calculated from NGS data (coefficient of determination R^2^ = 0.909, Figure S3), confirming the accuracy of our SNP frequency data.

### Analyses of population genome

**Figure 3:**
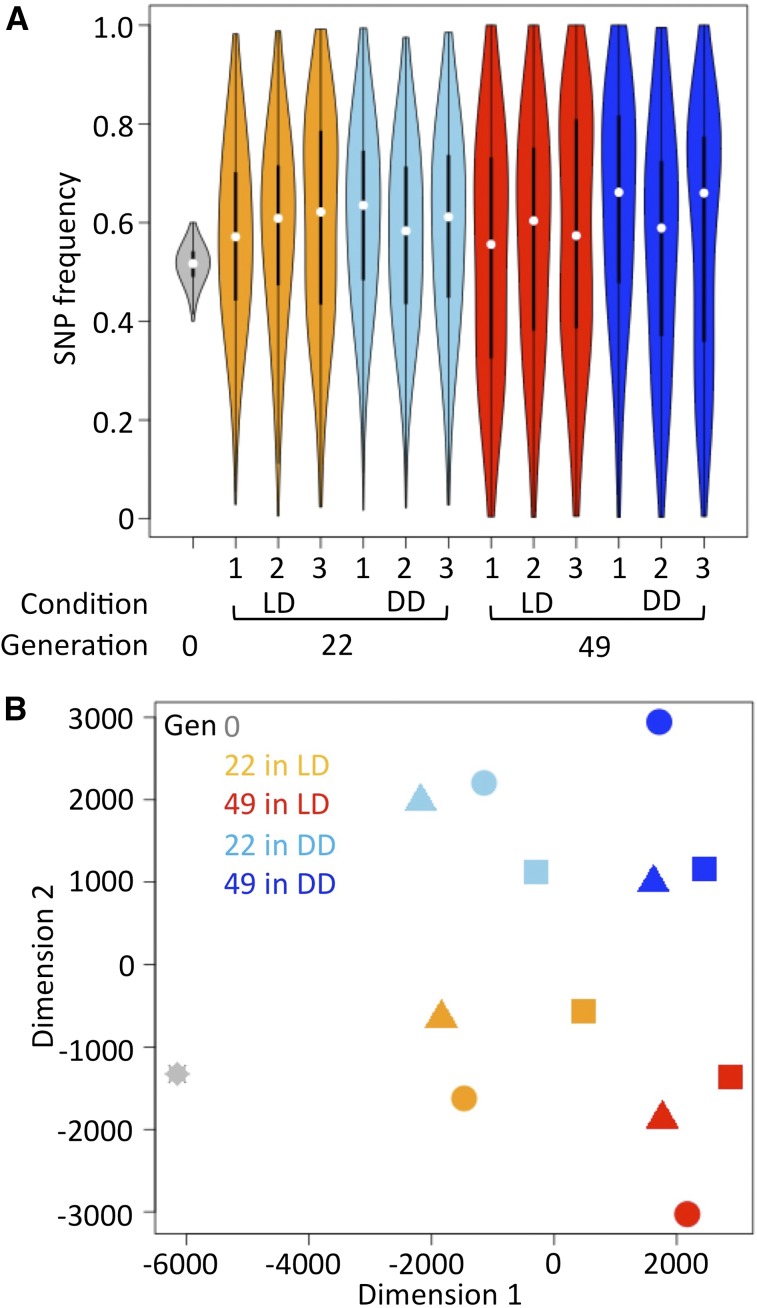
Frequency of Dark-fly’s SNPs in the LD- and DD-reared populations. (A) Violin plot of the frequency of Dark-fly’s SNPs in the populations reared in LD and DD conditions at generations 0, 22, and 49. White points and back thick bars represent median values and interquartile ranges of data, respectively. (B) MDS analysis of the overall SNP frequency. Profiles of SNP frequency in each replicate population were plotted in two dimensions using MDS analysis (stress = 15.9). Dimension one divided populations at different time points, and dimension two divided populations in LD and DD conditions.

We characterized the features of the overall SNP frequency in the populations, and observed that the SNP frequency became progressively more diverse during successive generations (comparing generations 0–22 and 22–49), as expected (Figure 3A). The average overall SNP frequency was not markedly different between populations (Table S5), but was slightly higher in the DD-reared population than in the LD-reared population at generation 49 (0.588 *vs.* 0.560 for the average of three replicates). Plotting the SNP frequency along the chromosomal position (Figure S4) revealed that the frequency of SNPs increased or decreased in position-dependent manners, but was roughly conserved among populations. The plots of SNP frequency displayed overall a “belt-like” pattern, with the only exception being around the centromere of chromosome 2: the discontinuity of SNP frequency in this region might be due to structural variants of this chromosome (see *Discussion*). These observations of SNP frequency suggest that some of Dark-fly’s SNPs would be advantageous, and some would be disadvantageous, so that their frequencies would increase or decrease, respectively, during successive generations.

To characterize the overall SNP frequency of successive populations reared under LD and DD conditions, we performed nonmetric MDS analysis of SNP frequencies. Figure 3B shows that the SNP frequencies showed a similar profile among replicate populations, and that the profile shifted from generation 22 to 49. Importantly, dimension two of the plot showed a clear difference between the LD- and DD-reared populations. These results indicate that the overall SNP frequency was similar among replicate populations reared in the same condition, but was progressively altered between LD- and DD-reared populations during successive generations. We suggest that overall, the changes of SNP frequency were rarely caused by random genetic drift, but rather were mainly caused by selection forces and that the selections under LD and DD conditions were recorded in the population genome.

### Comparison of SNP frequency between LD and DD conditions in successive generations

We next sought to identify SNPs showing different frequency in LD- and DD-reared populations. The mean frequency of each SNP in replicate populations was obtained for comparison in subsequent analyses. Then, allele frequency changes (AFCs) between conditions (frequency in DD − frequency in LD) were calculated for each SNP at generations 22 and 49. The distribution of AFCs gradually became broader during successive generations, *i.e.*, the difference of the frequency of SNPs increased with increasing generations (Figure S5). AFCs at generation 49 were slightly shifted in the plus-direction, indicating that the number of the Dark-fly’s SNPs showing higher frequency in the DD than in the LD condition was larger than that of the Dark-fly’s SNPs showing the opposite trend. This biased AFC distribution suggests that the Dark-fly genome carries a large fraction of SNPs positively selected in the DD condition or negatively selected in the LD condition.

**Figure 4:**
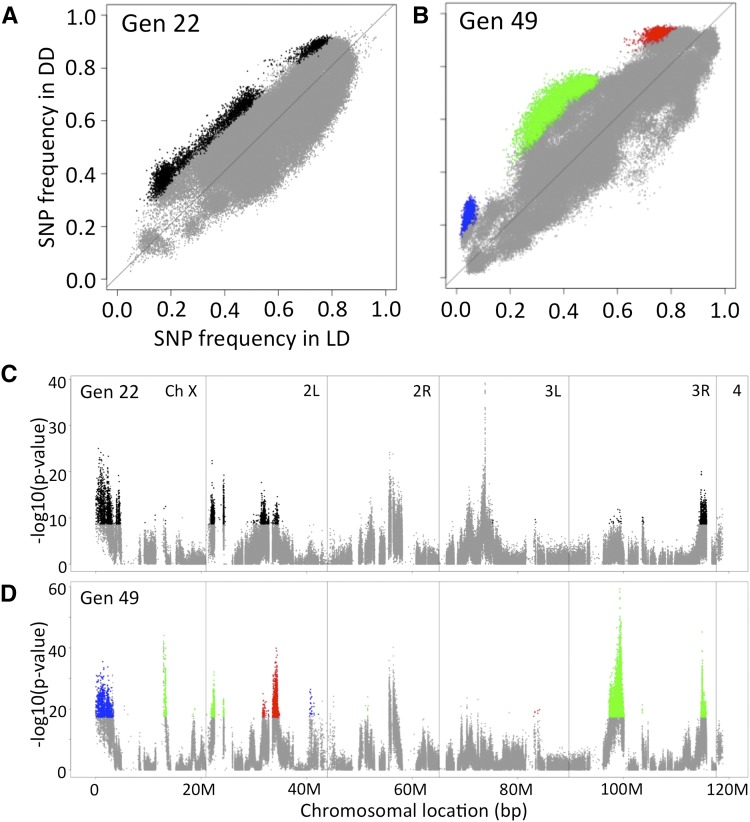
Comparison of SNP frequency in LD- and DD-reared populations. (A, B) Scatter plots comparing SNP frequency in LD and DD conditions at generation 22 (A) and 49 (B). The SNPs showing a significant difference of frequency in Fisher’s exact test (top 5% of p-values) and higher frequency in the DD than in the LD condition were colored black at generation 22 and red, green, and blue (type 1, type 2, and type 3, respectively) at generation 49. Other SNPs were colored gray. (C, D) P-values (Fisher’s exact test) for each SNP were plotted as reverse logarithm values along chromosomal position at generation 22 (C) and 49 (D). Colors corresponded to those shown in (A, B).

Next, we examined how the SNP frequencies were affected by light condition. For this, we plotted the frequency of each SNP in the LD condition (*x*-axis) and DD condition (*y*-axis) (Figure 4, A and B). Many SNPs showed the same frequency in the LD and DD conditions, and thus were plotted near the *y* = *x* line. However, some SNPs showed a different frequency in the LD and DD conditions, and thus were scattered far from this line. Compared to the results of generation 22, more SNPs showed differential frequency at generation 49. Fisher’s exact test was then performed to identify the SNPs showing significantly different frequency between the LD- and DD-reared populations, and identified 12,141 such SNPs at generation 22 and 31,704 such SNPs at generation 49 (Bonferroni-corrected p-value < 0.01). Among them, the number of SNPs showing higher frequency in DD than in LD was significantly larger than the number showing lower frequency (chi-square test, p-value = 1.9E-5 at generation 22 and p-value < 2.2E-16 at generation 49), confirming that a large fraction of Dark-fly’s SNPs increased in frequency in DD conditions. We focused on SNPs that showed higher frequency in the DD than in the LD condition, and whose p-values were ranked in the top 5% (colored dots in Figure 4, A and B). At generation 49, this criterion of the p-value (p-value < 1e-17.3) was adequate for detecting outliers from SNPs with a linear distribution of expected values (Figure S6), though this criteria when applied at generation 22 (p-value < 1e-8.67) was not adequate for detecting such outliers, indicating that more SNPs had been selected in the population at generation 49 than at generation 22. At generation 49, 6015 such SNPs were grouped into three types according to their relative frequency in LD *vs.* DD conditions: type 1, 2, and 3 showed high, middle, and low relative frequency, respectively, in the DD compared to LD condition on average (red, green, and blue in Figure 4B, see *Materials and Methods*). These three different types might have resulted from different selection patterns of the respective SNPs by DD *vs.* LD conditions.

**Figure 5:**
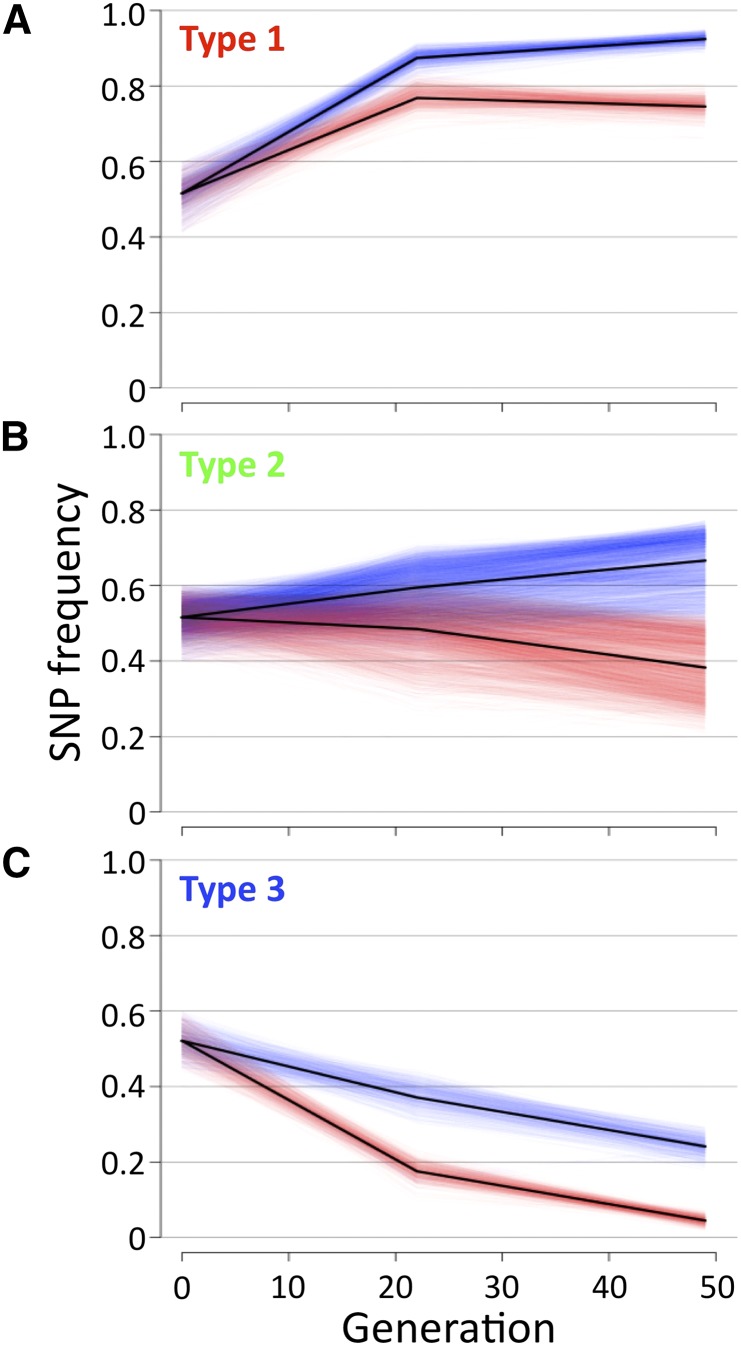
Trajectory of frequency of SNPs during successive generations. Temporal changes of SNP frequency were analyzed for each SNP type (A: type 1, B: type 2, C: type 3). Blue and red lines represent data of each SNP in DD- and LD-reared populations, respectively. Black lines represent mean SNP frequency of each type in DD- and LD-reared populations.

To further characterize the patterns of selection of these SNPs, we integrated the data of generations 0, 22, and 49, and examined the trajectory of SNP frequency through successive generations (Figure 5). Type 1 SNPs (Figure 4B, red, n = 1108) increased in frequency with increasing generations in both LD and DD conditions, but the increment was higher in the DD condition (Figure 5A). Fisher’s exact tests for the temporal changes of frequency indicated that a larger number of SNPs showed significant increments of frequency in the DD than in the LD condition (data not shown). These findings imply that type 1 SNPs were selected positively in both conditions, but the positive selection was stronger in the DD condition. Likewise, type 2 SNPs (Figure 4B, green, n = 4000) were selected positively in the DD but negatively in the LD condition (Figure 5B). Finally, type 3 SNPs (Figure 4B, blue, n = 907) were selected negatively in both conditions, but the negative selection was stronger in the LD condition (Figure 5C). These results suggested that there were at least three types of environmental selection acting on the Dark-fly genome.

We then examined the chromosomal positions of these selected SNPs, and found that the SNPs were accumulated in several distinct chromosomal regions (Figure 4D). The accumulation of many SNPs in a particular locus implies that one or a few SNPs in each locus would be truly under selection force, and other nearby SNPs in that locus would change their frequencies as a result of the effects of linkage (see also Figure S4). However, we found that SNPs categorized into the same selection type were mapped to several separate loci on different chromosomes. That is, several loci shared the same trajectory of selection, and thus we speculate that several responsible SNPs (genes) at separate loci might function together for an adaptive trait of Dark-fly (see *Discussion*).

### Candidates for adaptive genes

To search for a link between selected SNPs and Dark-fly’s traits, we further characterized the chromosomal loci carrying selected SNPs. We identified 28 such regions, which had accumulated selected SNPs and are separated from each other by at least 100 kb (Table S6). In our previous genome analyses, we identified ROH regions in the Dark-fly genome (Izutsu *et al.* 2012). An ROH region is a genomic region with homozygosity extending for a long distance, and is known as a signature of positive selection during a population’s history (McQuillan *et al.* 2008). We found that four out of the 28 regions selected in mixed populations overlapped with ROH regions (Table S6). This overlapping could not be explained merely by coincidence, because ROH regions cover only a much smaller fraction (3.5%) of the whole genome. Rather, the overlapping regions had been selected both in the history of Dark-fly and in the current experiment of selection in mixed populations.

Among the genes located in the 28 selected regions, we identified 1283 genes carrying SNPs or InDels in the Dark-fly genome (see *Materials and Methods*). The nonsynonymous SNPs and InDels located in the gene coding regions might alter the function and activity of gene products. Alternatively, the SNPs and InDels located in intergenic regions might affect the expression of genes. We consider these 1283 genes to be primary candidates selected in the mixed populations (File S1, File S2, and File S5).

**Figure 6:**
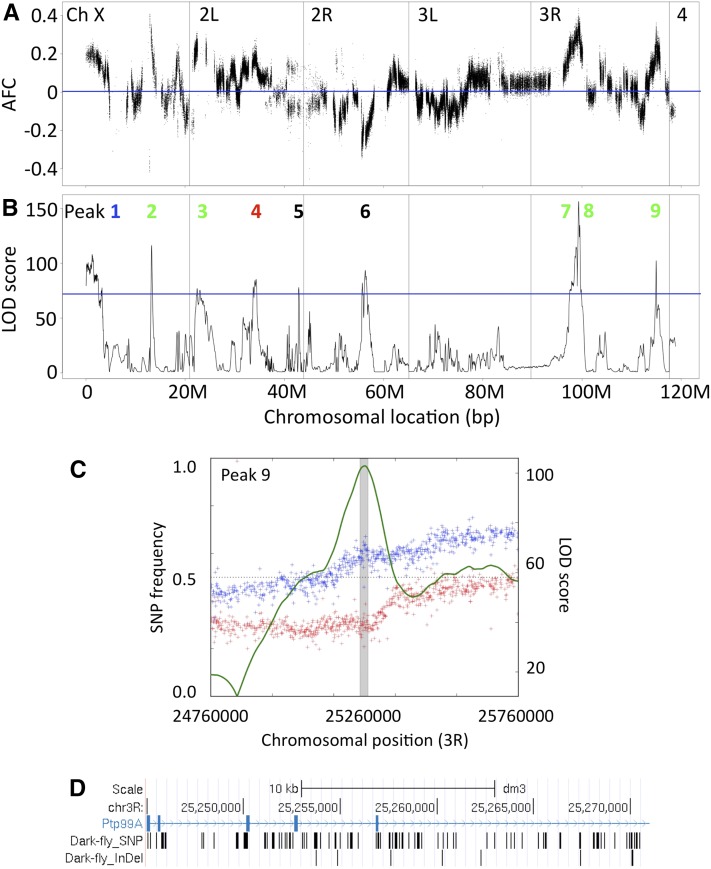
Chromosomal regions selected in the mixed populations. (A) AFC of each SNP (frequency in DD minus frequency in LD) at generation 49 were plotted along chromosomal position. A blue line represents AFC = 0, meaning equal frequency in LD- and DD-reared populations. (B) LOD scores of 1-kb windows at generation 49 were plotted along chromosomal position. The blue line represents the threshold (75) for detecting LOD peaks. Numbers in the graph indicate LOD peak number. (C) A magnified view of LOD peak nine. Red points indicate SNP frequency in LD and blue points indicate that in DD. The green line and gray bar represent LOD score and 90% credible interval span, respectively. (D) A view of UCSC Genomic Browser around the 90% credible interval span of LOD peak nine. Position of Dark-fly’s SNPs and InDels are represented by bars.

The 28 selected regions we identified constitute a total of 10,856,126 bases (6.4% of the genome), and include more than 1000 genes, so it would be laborious to test the role of each of these candidate genes for adaptive traits of Dark-fly. Thus, to narrow down the candidates, we utilized the MULTIPOOL program (Edwards and Gifford 2012), which predicts QTL in bulked segregant analysis. Comparing the LD- and DD-reared populations, the LOD scores at 1-kb windows were computed along the chromosome from the data of generations 22 and 49. We set an optional threshold of LOD score as 75, for extracting a clear difference between generation 22 and 49 (Figure S7). Using this threshold, we identified nine peaks of LOD scores in the data of generation 49 (Figure 6B). The LOD peaks detected were well correlated with the regions with high values of absolute AFCs, meaning that there was a large difference in these regions between the LD- and DD-reared populations (Figure 6, A and B). Among the nine LOD peaks, seven peaks showed a higher SNP frequency in the DD than in the LD condition, and were narrowed to a span (26–131 kb) with a 90% credible interval (Figure 6C and Figure S8). We listed 84 genes carrying SNPs and InDels within these LOD peaks (Table 2). For example, peak 9 (spanning for 26 kb) was located within a large gene, Ptp99A, encoding a protein tyrosine phosphatase (Figure 6, C and D). These 84 genes are candidates for genes associated with Dark-fly’s traits (File S3, File S4, and File S5, see *Discussion*), and we need to examine the roles of these genes in future work.

**Table 2:** Candidate regions identified at LOD peaks

LOD Peak No.	Chromosome	Peak Position	Credible Interval Start	Credible Interval End	Span (kb)	Genes	Selection Type
1	X	1252000	1237000	1339000	102	CG11417, CG11418, CG11448, CG12773, CG14770, CG14773, CG3056, CG32813, CG3719, SNF1A, futsch, png	3
2	X	13237000	13211000	13250000	39	CG15747, IP3K2, Jafrac1, RpS15Aa	2
3	2L	1515000	1476000	1559000	83	CG14351, CG18131, CG18132, CG31661, CG31926, CG31928, CG33128, CG7420, Or22a, Or22b, halo	2
4	2L	13159000	13316000	13438000	122	CG10859, CG16826, CG16848, CG16956, CG16957, CG16970, CG31855, CG6523, CG6565, CG7099, CG7110, CG9293, CG9302, CG9305, CG9306, CG9377, CG9395, Nnp-1, RpL24, Tap42, Tehao, Vm34Ca, beta’Cop, loqs	1
7	3R	9109000	9061000	9192000	131	Ace, CG11686, CG15887, CG15888, CG32473, CG8449, CG8630, CG8773, CG8774, CG8784, CG8790, CG8795, CheA87a, Lip3, Osi22, Ravus, Su(var)3-7, mthl12, poly, wntD	2
8	3R	9608000	9601000	9638000	37	CG42375, CG9286, CG9288, CG9297, Cht5, Dip-B, tRNA:CR31331, tRNA:CR31588, tal-1A, tal-2A, tal-3A, tal-AA	2
9	3R	25260000	25245000	25271000	26	Ptp99A	2

Seven candidate regions showing significant difference in SNP frequency between LD and DD conditions and high score of LOD. Chromosome, positions, start and end of 90% credible interval, span, genes, and selection types (Figure 5) are given for each region.

## Discussion

### The adaptive traits of Dark-fly in a dark environment

Here we found that the relative fitness of Dark-fly under reproductive competition against other strains of flies was higher in the DD compared to the LD condition. Various wild-type lines we examined did not show such an advantage in the DD condition, indicating that Dark-fly is a unique line with regard to its display of reproductive success in a dark environment. Reproductive success could be increased via various traits, such as mating behaviors, egg-laying ability, egg-to-adult viability, and so on. From the results of our reproductive competition experiments between Dark-fly and the Oregon-R-S strain, we found that the proportion of Red progeny (resulting from Dark-fly females and DsRed-Oregon-R-S males) was decreased in the DD condition, whereas the proportion of White progeny (resulting from Dark-fly females and Dark-fly males) tended to be increased in this condition. There are various possible explanations for this result. One possibility is that Dark-fly females might prefer Dark-fly males as partners, and avoid DsRed-Oregon-R-S males, in the dark. Regarding such a preference, Dark-fly females might detect some specific cues, such as odors or sounds, to discriminate partners in the absence of visual cues (also see below).

When we performed the competition assay against Urbana-S competitors, we observed a different pattern of performance of Dark-fly. That is, the proportion of White progeny was increased in the DD condition, whereas that of Green progeny (from GFP-Urbana-S females and Dark-fly males) was decreased. In this case, Dark-fly males might have a preference for Dark-fly females compared to Urbana-S females in the dark. Thus, Dark-fly exhibited different advantages when reared in the dark depending on the competitor line. Although we could not define particular traits contributing to Dark-fly’s fitness in the dark, we confirmed here that Dark-fly possesses some advantageous traits for reproduction in a dark environment, and these advantages suggest that Dark-fly could dominate over the wild-type fly in the dark.

### Mixed population experiment

We designed a mixed population experiment to identify genes involved in the adaptation of Dark-fly. In many previous studies about environmental adaptation, QTL analyses revealed the genes associated with traits of organisms (Nadeau and Jiggins 2010). Most of those studies focused on unique traits, such as morphological features, presumably related to the adaptation. Although Dark-fly does not possess any obvious morphological traits, it exhibits some advantages regarding reproduction in the dark. Therefore, we carried out experiments aiming at fitness-based selection of genes in a mixed population of Dark-fly and other fly strains, and as a result succeeded in observing environment-dependent selections of some genes (although we might have overlooked some weak selections using this approach).

GWAS is one of the approaches for analyzing population genomes with large genetic diversity in their background. Its statistical power is very high for detecting the consistency of genome alterations across large populations. Since our mixed population experiment started from two fly strains, the genome diversity in the population was quite low compared to that in GWAS. However, our experiment has the advantage that it uses a defined environment for the selection and makes it possible to observe the temporal pattern of the selection during successive generations. The mixed population experiment is thus a powerful strategy for identifying genome variations related to the fitness in a defined environment, and could be useful for application to other organisms.

### Genome analysis of mixed populations

We analyzed the SNP frequency in the pooled genome of the populations. Analysis of pooled genomes has now become widely used because of its cost-effective high performance (Schlotterer *et al.* 2014). Although the pooled genome loses the information of the haplotype structure of the population, it deals with a large population size and the whole genome at once. We extracted genomic DNA from 1000 individual flies, and obtained sequence data covering 179×-depth of the genome on average. We analyzed more than 140,000 SNPs, and thus obtained fine-scale information along the whole genome (at approximately 1-kb intervals on average). From these data, we characterized the genomes of the large populations.

For this population genome analysis, we used confirmed Dark-fly SNPs (Figure S2, the black-outlined area), which were identified as Dark-fly’s SNPs in our previous study (Izutsu *et al.* 2012) and showed a frequency of 0.4–0.6 in the mixed population at 0 generation. It should be noted, however, that we detected 161,893 SNPs which showed a frequency of 0.4–0.6 at 0 generation but were identified in neither Dark-fly nor Oregon-R-S in our previous study (Figure S2). We might have overlooked these SNPs in the previous analysis, probably because of low coverage of sequencing. In addition to such hidden SNPs, we did not evaluate heterozygous SNPs of the original Dark-fly in this study. Although we detected a small number of heterozygous SNPs in the previous analyses of the Dark-fly genome, we might have overlooked more such SNPs. In this study, we used only confirmed homozygous Dark-fly SNPs to examine selections, although as a result we might have missed some SNPs actually selected. In order to minimize such an oversight, we will need to obtain more precise and comprehensive genome data for parental Dark-fly. Thus, improved accuracy of the sequence of the parental genome would be an important factor for ensuring the analytical scale and precision in the pooled genome analysis.

The SNP frequency in the population would be affected by selective pressure and by recombination in the genome, and consequently the frequency of SNPs would change in a coordinated manner along chromosomal positions. However, we observed marked discontinuity of the SNP frequency in the region near the centromere of chromosome 2 (Figure S4). This was probably not due to accidental extraction of some SNPs in a population, because replicate populations showed almost the same zigzag pattern of frequency changes along this chromosomal region. Rather, we speculate that there might be structural variants (duplication, deletion, inversion, insertion, *etc*.) of the Dark-fly or Oregon-RS genome in this region that might cause discontinuous changes of SNP frequency. Our analysis did not evaluate SNP selection precisely in this region, and we had to eliminate this region for identifying adaptive SNPs. Such structural variants of chromosomal regions would be a confounding issue for analyzing population genomes, and detailed information about the parental genome would be required for resolving the issues presented by such variants.

### SNP frequency trajectory during selection

We detected SNPs showing different frequencies between the LD and DD-reared populations. We unexpectedly detected some Dark-fly SNPs showing higher frequency in the LD compared to the DD condition (see Figure 6A, minus values of AFC). Dark-fly might possibly carry such SNPs advantageous in LD as a result of random genetic drift during its history. Alternatively, it is possible that these SNPs are neutral but that their frequencies in the mixed populations were changed by random genetic drift. However, we emphasize that a much larger fraction of Dark-fly’s SNPs increased in frequency in the DD condition (higher in DD: 6015 SNPs; higher in LD: 1129 SNPs). This implies that SNP selection was not totally random, but rather mostly biased to dark-selection. We therefore suggest that unique features of Dark-fly supporting condition-dependent fitness largely result in the difference of SNP frequency in the mixed population in LD and DD conditions.

From analyses of the population genome at generation 49, we identified Dark-fly SNPs showing higher frequency in the DD than in the LD condition, and categorized them into three types according to overall frequency: type 1, 2, and 3 showed high, middle, and low frequency, respectively. We also observed the time-course trajectory of the SNP frequency through successive generations for each type, and found apparently different selections acting on each type. From the overall trajectory of their increasing frequency across successive generations, we suggest that the type 1 SNPs were advantageous and positively selected in the DD condition. From the overall trajectory of decline, type 3 SNPs appeared to be disadvantageous and negatively selected in the LD condition. Type 2 SNPs appeared to be advantageous in DD and simultaneously disadvantageous in the LD condition, so they were selected positively in DD and negatively in LD. Thus, Dark-fly carries at least three types of SNPs undergoing different modes of selection in the dark.

It is possible that SNPs showing a similar trajectory might be associated with the same trait of Dark-fly. In order to address the possibility, we will need to identify the genes responsible for a Dark-fly trait and to examine the epistatic interactions of several genes in the future. From the present analyses of SNP frequency trajectory, we suggest that Dark-fly possesses at least three traits differentially selected in dark conditions, and that a number of genes contribute to each trait.

### Candidates for Dark-fly adaptive genes

The selected SNPs were accumulated in several chromosomal loci. It is unlikely that all of those SNPs contribute to an advantage or disadvantage in DD or LD conditions. Instead, one or a few SNPs in each locus were probably selected positively or negatively, and the frequencies of the other SNPs were changed as a result of the effects of linkage. It is also possible that InDels or other structural variants in the locus might contribute to the Dark-fly’s traits. In this case, the SNPs would simply be markers for detecting the locus.

In general, there are two possible effects of mutations (SNPs, InDels, or structural variants) that affect unique traits of organisms. First, these mutations might be located within the coding region of a gene and might generate nonsynonymous or frameshift mutations in the gene. In this case, the mutations would alter the activity of gene products. Second, the mutations might be located in an intergenic region, and might alter the expression of a gene(s). Both cases indeed have been documented in previous studies of evolved traits of organisms (Pal *et al.* 2006; Nadeau and Jiggins 2010). One way to distinguish between these possibilities is transcriptome analysis. We have performed transcriptome analysis for the wild-type fly and Dark-fly reared in LD and DD conditions and have detected some differentially expressed genes (N. Fuse, M. Izutsu, and K. Agata, unpublished data); we will report the results in the near future.

As primary candidates for Dark-fly’s adaptive genes, here we identified 1283 genes that are located in selected chromosomal regions and that carry SNPs or InDels in the Dark-fly genome (Figure 4D and Table S6). We used the MULTIPOOL program, a program that has successfully identified QTL loci in previous studies, and that enabled us to narrow down the candidates to seven selected regions, and to identify 84 genes carrying SNPs or InDels in these regions of the Dark-fly genome. We suggest that these genes are potentially strong candidates for those involved in Dark-fly’s adaptation.

Concerning the reproductive success of Dark-fly in a dark environment, as shown in Figure 1, we considered several hypotheses about possible roles of candidate genes. Among the candidates (Table 2), Or22a, Or22b, and CheA87a encode olfactory and chemosensory receptors. Although the chemical entities binding to these receptors have not been identified yet, it is possible that these receptors might perceive pheromonal signals. We speculate that the mutations of pheromone receptors might enhance sexual activity in Dark-fly. Other notable candidate genes are CG8784 and CG8795 (also called PK2-R1 and -R2), encoding receptors for a neuropeptide, Pyrokinin-2 (also called Pheromone Biosynthesis Activating Neuropeptide; PBAN), and it is known that this humoral signaling activates pheromone synthesis in several *Diptera* species (Choi *et al.* 2001; Rosenkilde *et al.* 2003). We also note that Ptp99A, encoding a protein tyrosine phosphatase, is known to be involved in olfactory memory formation (Walkinshaw *et al.* 2015). Thus, these candidate genes might be related to unusual pheromone synthesis and/or perception in Dark-fly.

We also considered another hypothesis, namely, that Dark-fly might possess abnormal circadian rhythm that results in higher fitness in the dark. While Dark-fly retains circadian locomotor rhythms (Imafuku and Haramura 2011) as noted in the *Introduction*, its rhythms of mating behavior and gametogenesis physiology have not been investigated. Some genes in the candidate list are indeed expressed in circadian oscillatory manners (Ace and Dip-B; Hooven *et al.* 2009; Claridge-Chang *et al.* 2001). The mouse homolog of SNF1A (also called AMPKα) is known to regulate the circadian clock through cryptochrome phosphorylation (Lamia *et al.* 2009). Another notable candidate, Jafrac1, is an evolutionarily conserved marker for circadian rhythms (Edgar *et al.* 2012). In the future, we will further analyze the functions of these candidate genes identified here to elucidate the molecular mechanisms underlying the environmental adaptation of Dark-fly.

## Supplementary Material

Supporting Information
