# Supplementary material for: Dynamics of Dark-Fly Genome Under Environmental Selections
Source: G3 (Bethesda). 2015 Dec 4;6(2):365–76. doi: 10.1534/g3.115.023549 (PMC4751556; doi:10.1534/g3.115.023549)
Supplement: Supporting Information [file supp_6_2_365__index.html]

Dynamics of Dark-Fly Genome Under Environmental Selections — Supporting Information 

# Dynamics of Dark-Fly Genome Under Environmental Selections

## Supporting Information for Izutsu *et al.*, 2016

**Files in this Data Supplement:**

- Figure S1 - The proportion of progeny in the fitness assay in each replicate test. (.pdf, 1,255 KB)
- Figure S6 - Characterization of p-values of Fisher?s exact test. (.pdf, 745 KB)
- Figure S7 - Characterization of LOD scores. (.pdf, 563 KB)
- Figure S8 - LOD peaks. (.pdf, 2,923 KB)
- Table S1 - Primer sets for measuring SNP frequency by qPCR. (.pdf, 78 KB)
- Table S2 - Comparative fitness of tester lines under mating competition assay. (.pdf, 72 KB)
- Table S3 - Statistical test of differences of progeny proportions in mating competition assay. (.pdf, 69 KB)
- Table S4 - Population size of mixed populations. (.pdf, 67 KB)
- Table S5 - Average SNP frequency in populations. (.pdf, 69 KB)
- Table S6 - Selected regions identified using Fisher's exact test. (.pdf, 72 KB)
- File S5 - Supporting information legends for Files S1-S4.
- Figure S2 - Comparison of SNPs identified in the mixed population genome and in the previous genome sequencing. (.pdf, 697 KB)
- Figure S3 - Reliability of SNP frequency data. (.pdf, 637 KB)
- Figure S4 - SNP frequency along chromosomal position. (.pdf, 2,595 KB)
- Figure S5 - Histogram of allele frequency change (AFC) between LD and DD populations. (.pdf, 373 KB)
- File S1 - Effects of SNPs located in selected regions. (.zip, 511 KB)
- File S2 - Effects of InDels located in selected regions. (.zip, 14 KB)
- File S3 - Gene ontology terms of 84 candidate genes. (.zip, 7 KB)
- File S4 - Non-synonymous SNPs in candidate genes. (.zip, 2 KB)
