# Supplementary material for: Dynamics of Dark-Fly Genome Under Environmental Selections
Source: G3 (Bethesda). 2015 Dec 4;6(2):365–76. doi: 10.1534/g3.115.023549 (PMC4751556; doi:10.1534/g3.115.023549)
Supplement: Supporting Information [file supp_g3.115.023549_FigureS1.pdf]

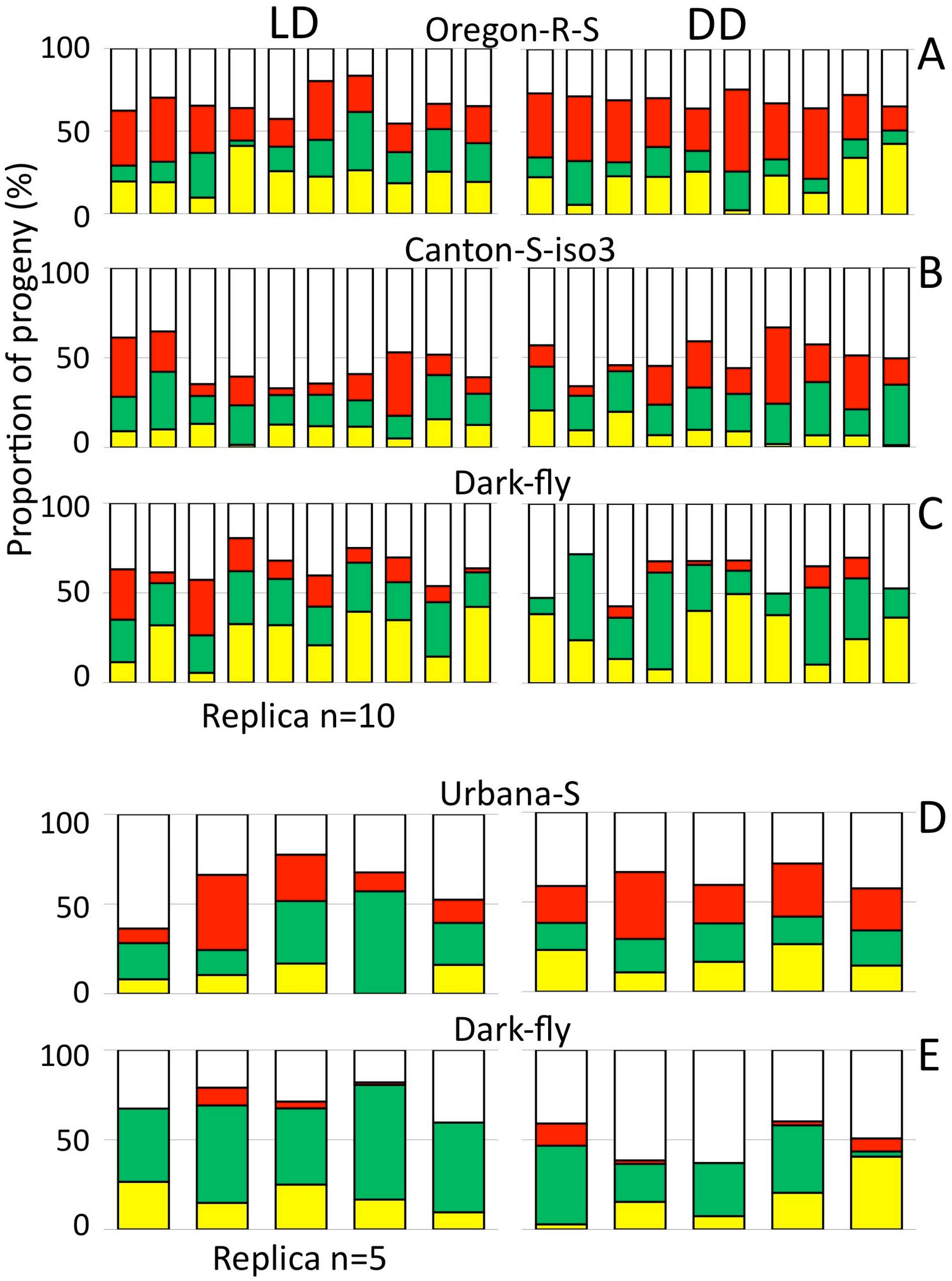

Figure S1 The proportion of progeny in the fitness assay in each replicate test

Raw data of proportion of progeny are shown for each replicate. The deviations of the data were relatively large, but the proportion of Dark-fly progeny was significantly different between the LD (left) and DD (right) conditions. (A-C) Assays against the Oregon-R-S competitors. (D, E) Assays against the Urbana-S competitors. Tester lines are indicated on the top of the graph.
