# Supplementary material for: Dynamics of Dark-Fly Genome Under Environmental Selections
Source: G3 (Bethesda). 2015 Dec 4;6(2):365–76. doi: 10.1534/g3.115.023549 (PMC4751556; doi:10.1534/g3.115.023549)
Supplement: Supporting Information [file supp_g3.115.023549_FigureS6.pdf]

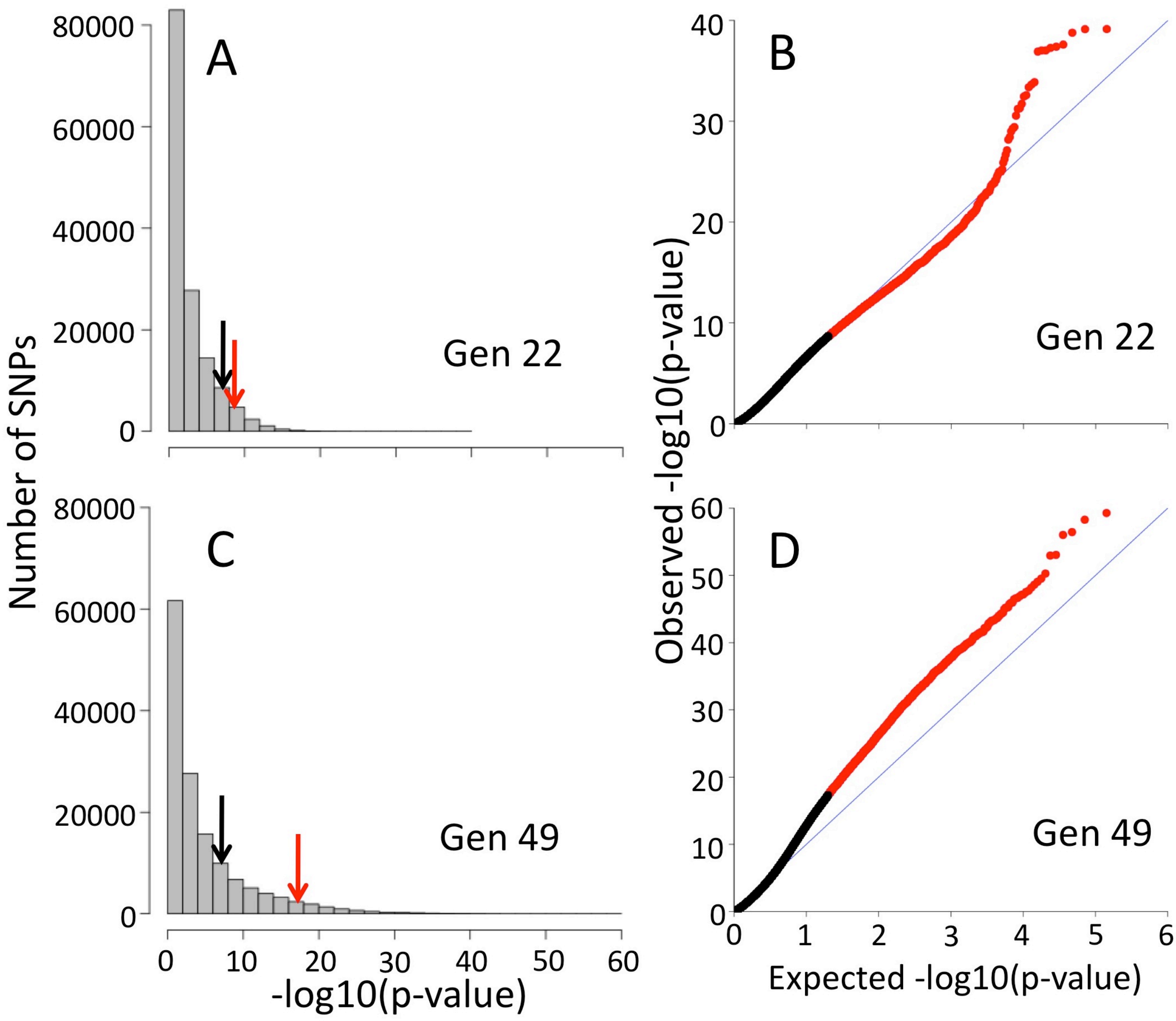

Figure S6 Characterization of p-values of Fisher's exact test

(A, C) Histogram of p-value calculated using Fisher's exact test at generation 22 (A) and 49 (C). Black arrows indicate Bonferroni-corrected p-value < 0.01 and red arrows indicate top 5% p-values. (B, D) QQ-plot of p-values (reverse logarithm value of theoretical and actual p-values). At generation 22 (B), top 5 % of the p-values (red dots) were started from the linear distribution of p-values (on the diagonal line: blue). At generation 49 (D), the top 5 % of p-values were detected outside of the linear distribution of p-values. Many p-values (below about 1e-10) were shifted from the starting linear distribution, probably due to the effects of genetic linkage between many SNPs.
