# Supplementary material for: Dynamics of Dark-Fly Genome Under Environmental Selections
Source: G3 (Bethesda). 2015 Dec 4;6(2):365–76. doi: 10.1534/g3.115.023549 (PMC4751556; doi:10.1534/g3.115.023549)
Supplement: Supporting Information [file supp_g3.115.023549_FigureS7.pdf]

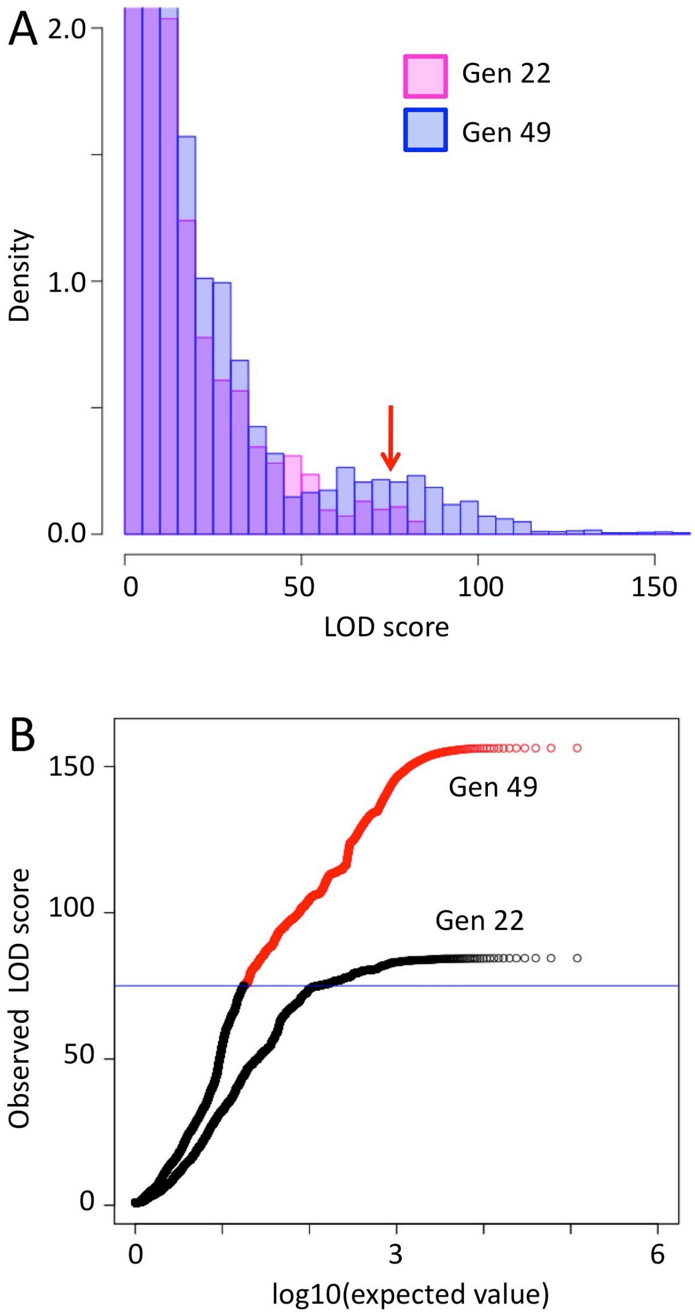

Figure S7 Characterization of LOD scores

(A) Histogram of LOD scores. Red and blue bars indicate density of LOD scores of generation 22 and 49 populations, respectively. The red arrow indicates an optional threshold of LOD score (= 75) to detect LOD peaks. (B) QQ-plot of LOD scores of generation 22 and 49 (logarithm value of theoretical and actual values). The blue line indicates the threshold of actual LOD score, and red circles indicate LOD scores of generation 49 above the threshold.
