# Supplementary material for: Dynamics of Dark-Fly Genome Under Environmental Selections
Source: G3 (Bethesda). 2015 Dec 4;6(2):365–76. doi: 10.1534/g3.115.023549 (PMC4751556; doi:10.1534/g3.115.023549)
Supplement: Supporting Information [file supp_g3.115.023549_FigureS8.pdf]

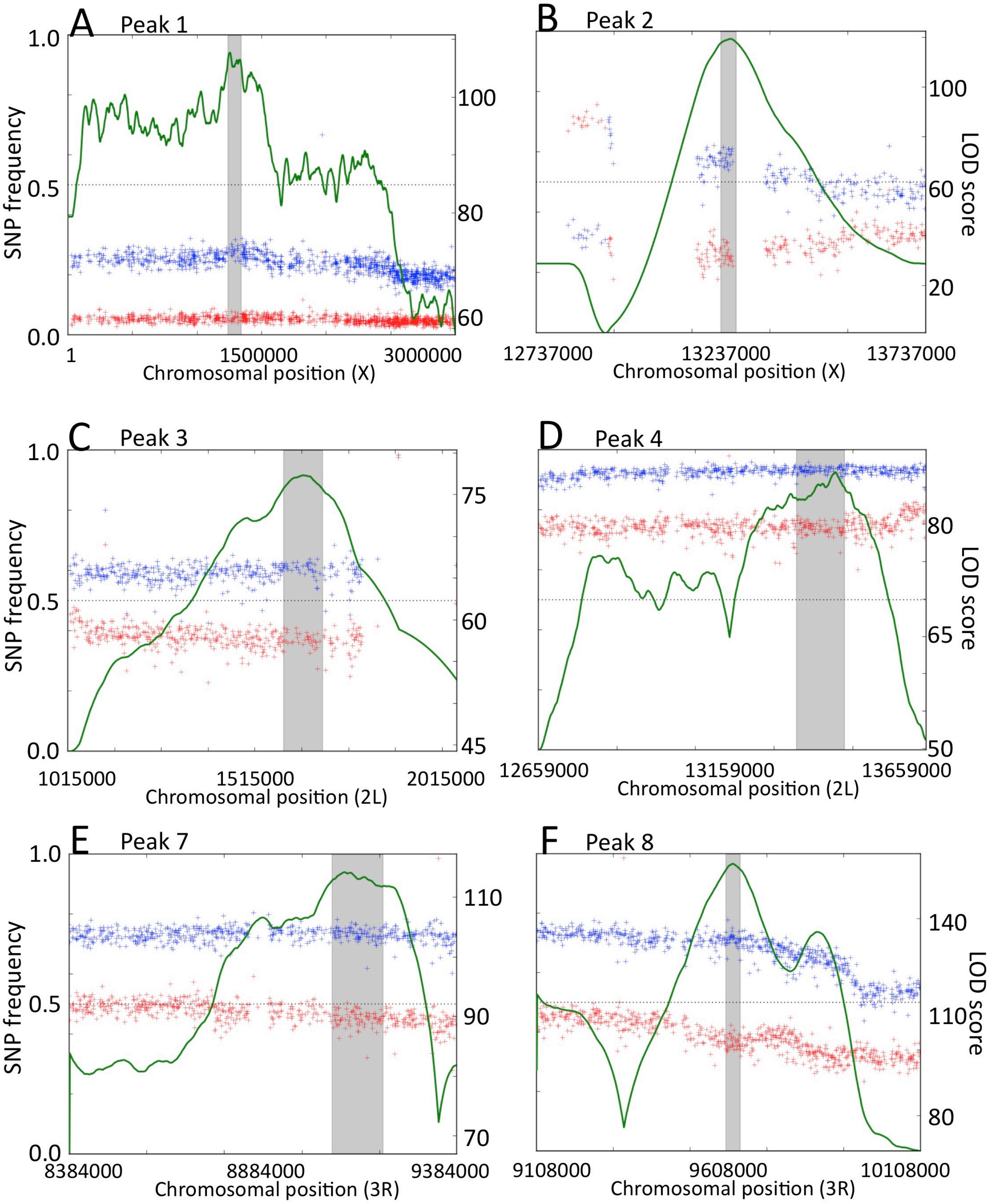

Figure S8 LOD peaks

Red and blue points indicate SNP frequency in LD and DD, respectively. Green lines indicate LOD scores, and gray bars indicate 90% credible interval span. (A) peak 1, (B) peak 2, (C) peak 3, (D) peak 4, (E) peak 7, and (F) peak 8 (see Figure 6B).
