# Supplementary material for: Dynamics of Dark-Fly Genome Under Environmental Selections
Source: G3 (Bethesda). 2015 Dec 4;6(2):365–76. doi: 10.1534/g3.115.023549 (PMC4751556; doi:10.1534/g3.115.023549)
Supplement: Supporting Information [file supp_g3.115.023549_TableS1.pdf]

Table S1 Primer sets for measuring SNP frequency by qPCR

The chromosomal position, reference allele, Dark-fly allele and nucleotide sequences of primers are shown. The reverse primer was common for combinations with Oregon-R-S (ORS)- and Dark-fly (D)-type primers.

SNP location		Ref	SNP	SNP type	Sequence (5'-3')
Chrom	Position				
X	637, 691	G	C	ORS	GTACTGGGTAAACGGCACCAAGC
				D	GTACTGGGTAAACGGCACCAAGG
				reverse	GTTGTTCTGCAGTTCACTCCGGG
X	4, 442, 501	C	G	ORS	CTGGCTGGGATTGGGCTATTG
				D	CTGGCTGGGATTGGGCTATTC
				reverse	TTTACGGAGGACGGGGATGTG
2L	1, 166, 904	C	T	ORS	CCTGTTCCGTTCGAGTTTTG
				D	CCTGTTCCGTTCGAGTTTTA
				reverse	AGATGTTGACCTGATGACTG
				ORS	GGTTGTTGAGTTACTCACTA
2L	3, 400, 117	T	C	D	GTTGTTGAGTTACTCACTG
				reverse	GGTTATGGTTTGTGGCATTT
2R	12, 478, 534	G	A	ORS	CTCTGAAGGAGACCACCAACATC
				D	CTCTGAAGGAGACCACCAACATT
				reverse	TTCCAGTTCCTTGAGGGCATCAT
3L	8, 723, 043	C	T	ORS	CGCTGTAAGTGTCCCTGAGAACCGC
				D	CGCTGTAAGTGTCCCTGAGAACCGT
				reverse	GGAGCTATCCTGACCGTAAGATCGC
3R	14, 053, 888	T	G	ORS	GCCTCCTGGATGCGCACGGTA
				D	CCTCCTGGATGCGCACGGTC
				reverse	TGGTGCGTGCCCGTGCCTTCA
3R	24, 169, 333	C	T	ORS	CTTGCTGGTCGATGGCTCCACTCTG
				D	CCTTGCTGGTCGATGGCTCCACTCTA
				reverse	GGGATGTGACTCCTGTAAAACGCCGC

3R	25, 087, 734	G	A	ORSD	CTCACACCACCCAATCCGACCC<br>CCTCACACCACCCAATCCGACCT
				reverse	GGTGCATTTTACCCCGCAGCCGT
