# Supplementary material for: Dynamics of Dark-Fly Genome Under Environmental Selections
Source: G3 (Bethesda). 2015 Dec 4;6(2):365–76. doi: 10.1534/g3.115.023549 (PMC4751556; doi:10.1534/g3.115.023549)
Supplement: Supporting Information [file supp_g3.115.023549_TableS2.pdf]

Table S2 Comparative fitness of tester lines under mating competition assay

Mean numbers of progeny counted in the mating competition are shown. P-values for comparison of the progeny number between LD and DD conditions were estimated by the Welch's t-test. Although some combinations of competitor and tester showed significant difference of progeny number, the differences were mostly independent on the differences in progeny proportions. Comparative fitness of tester lines were calculated as described in Materials and Methods. P-values for comparison of the comparative fitness between LD and DD conditions were calculated by the Mann-Whitney U test. *: p-value < 0.05, **: p-value < 0.01

Tester lines	Condi tion	Number of experi ments	Mean progeny number	Standard deviation of progeny number	p-value for progeny number	Comparative fitness of tester (%)	p-value for comparative fitness
vs Oregon-R-S competitor							
Oregon-R-S	LD	10	348	43.7	8.E-04**	55.18	6.3E-01
	DD	10	235	73.4		54.42	
Canton-S-iso3	LD	10	193	22.3	4.E-03**	72.12	3.9E-01
	DD	10	224	18.5		70.05	
Dark-fly	LD	10	141	27.7	6.E-01	54.12	8.0E-01
	DD	10	149	35.8		55.60	
vs Urbana-S competitor							
Urbana-S	LD	5	118	16.6	7.E-01	64.83	2.2E-01
	DD	5	126	20.6		59.41	
Dark-fly	LD	5	75	36.0	7.E-01	54.83	3.2E-02*
	DD	5	80	26.4		66.79	
