# Supplementary material for: Dynamics of Dark-Fly Genome Under Environmental Selections
Source: G3 (Bethesda). 2015 Dec 4;6(2):365–76. doi: 10.1534/g3.115.023549 (PMC4751556; doi:10.1534/g3.115.023549)
Supplement: Supporting Information [file supp_g3.115.023549_TableS3.pdf]

Table S3 Statistical test of differences of progeny proportions in mating competition assay

The proportions of progeny in the mating competition assay were compared between LD and DD conditions. P-values of the Mann-Whitney U test are shown for each progeny group (Yellow, Green, Red, and White). *: p-value < 0.05, **: p-value < 0.01

Competitor	Oregon-R-S			Urbana-S	
Tester	Oregon-R-S	Canton-S-iso3	Dark-fly	Urbana-S	Dark-fly
Yellow progeny	9.1E-01	2.7E-01	7.4E-01	1.5E-01	6.9E-01
Green progeny	1.5E-01	1.3E-01	1.0E+00	2.2E-01	3.2E-02*
Red progeny	6.3E-02	6.3E-01	6.2E-03**	4.2E-01	5.4E-01
White progeny	1.9E-01	2.8E-01	5.9E-01	1.0E+00	1.6E-02*
