# Supplementary material for: Dynamics of Dark-Fly Genome Under Environmental Selections
Source: G3 (Bethesda). 2015 Dec 4;6(2):365–76. doi: 10.1534/g3.115.023549 (PMC4751556; doi:10.1534/g3.115.023549)
Supplement: Supporting Information [file supp_g3.115.023549_TableS4.pdf]

Table S4 Population size of mixed populations

Mean population size for each mixed population estimated by measuring the weight of flies is shown (mean and standard deviation, n=58). There was no significant size difference between these populations (one-way ANOVA, p-value = 0.171).

Population name	Mean population size (No. of flies)	Standard deviation
L1	903.3	268.4
L2	967.8	320.5
L3	1028.8	314.7
D1	984.9	357.2
D2	1054.1	336.0
D3	970.2	324.0
