# Supplementary material for: Dynamics of Dark-Fly Genome Under Environmental Selections
Source: G3 (Bethesda). 2015 Dec 4;6(2):365–76. doi: 10.1534/g3.115.023549 (PMC4751556; doi:10.1534/g3.115.023549)
Supplement: Supporting Information [file supp_g3.115.023549_TableS5.pdf]

Table S5 Average SNP frequency in populations

Average frequency of 143,013 SNPs which were detected in all replicate populations is shown for the populations at generations 0, 22, and 49. The population name describes the conditions (L: LD, D: DD condition) + replicate ID number. The population at generation 0 was common for all replicates.

Population name	Generation 0	Generation 22	Generation 49
L1	0.51	0.57	0.53
L2		0.59	0.57
L3		0.60	0.58
D1		0.62	0.63
D2		0.57	0.56
D3		0.59	0.57
