# Supplementary material for: Dynamics of Dark-Fly Genome Under Environmental Selections
Source: G3 (Bethesda). 2015 Dec 4;6(2):365–76. doi: 10.1534/g3.115.023549 (PMC4751556; doi:10.1534/g3.115.023549)
Supplement: Supporting Information [file supp_g3.115.023549_TableS6.pdf]

Table S6 Selected regions identified using Fisher's exact test

Twenty-eight regions showed significant difference in SNP frequency between LD and DD conditions. Chromosome, positions, length, and number of SNPs are shown for each region. Some regions were overlapped with previously identified runs of homozygosity (ROH) regions (Izutsu et al., 2012).

Locus #	Chr	Position start base number	Position end base number	Number of SNPs	Length (bp)	ROH#
1	X	34469	3425312	879	3390844	
2	X	12923028	12935960	24	12933	
3	X	13150429	13443864	133	293436	
4	X	18354875	18354875	1	1	
5	X	18477432	18477432	1	1	
6	X	18775972	18775979	2	8	
7	2L	943188	1642473	242	699286	
8	2L	2449113	2449113	1	1	
9	2L	3287684	3441346	34	153663	ROH1
10	2L	10710873	10903073	28	192201	
11	2L	11008734	11442767	27	434034	
12	2L	11774233	11925517	5	151285	
13	2L	12596720	13943593	1042	1346874	ROH5
14	2L	19632949	19699386	5	66438	
15	2L	19810710	19906311	10	95602	
16	2L	20017229	20124894	6	107666	
17	2L	20472164	20514957	4	42794	
18	2L	21326181	21332720	2	6540	
19	2R	7639983	7639991	3	9	
20	3L	13282160	13282160	1	1	
21	3L	17922171	17922171	1	1	
22	3L	18143348	18143352	2	5	
23	3L	18686472	18686473	2	2	
24	3L	19029901	19029901	1	1	ROH15
25	3R	7489844	7489844	1	1	

26	3R	7636369	10459538	3081	2823170	ROH21
27	3R	13886468	13897474	5	11007	
28	3R	24977109	26005430	467	1028322	
