# Supplementary material for: Dynamics of Dark-Fly Genome Under Environmental Selections
Source: G3 (Bethesda). 2015 Dec 4;6(2):365–76. doi: 10.1534/g3.115.023549 (PMC4751556; doi:10.1534/g3.115.023549)
Supplement: Supporting Information [file supp_g3.115.023549_FileS5.pdf]

Supporting File legends

File S1 (5.4MB)

Effects of SNPs located in selected regions

Output file of snpEff to identify the genes affected by Dark-fly's SNPs located at the selected regions.

File S2 (90KB)

Effects of InDels located in selected regions

Output file of snpEff to identify the genes affected by Dark-fly's InDels located at the selected regions.

File S3 (41KB)

Gene ontology terms of 84 candidate genes

Gene ontology terms of candidate genes for “Biological process” and “Molecular function” were listed.

File S4 (4KB)

Non-synonymous SNPs in candidate genes

Non-synonymous SNPs in 84 candidate genes were searched from File 1. Some SNPs could affect multiple transcripts of one gene, and thus we excluded redundant effects of one SNP at the same location. We listed 52 non-synonymous SNPs in 30 candidate genes.
