# Supplementary material for: Dynamics of Dark-Fly Genome Under Environmental Selections
Source: G3 (Bethesda). 2015 Dec 4;6(2):365–76. doi: 10.1534/g3.115.023549 (PMC4751556; doi:10.1534/g3.115.023549)
Supplement: Supporting Information [file supp_g3.115.023549_FigureS2.pdf]

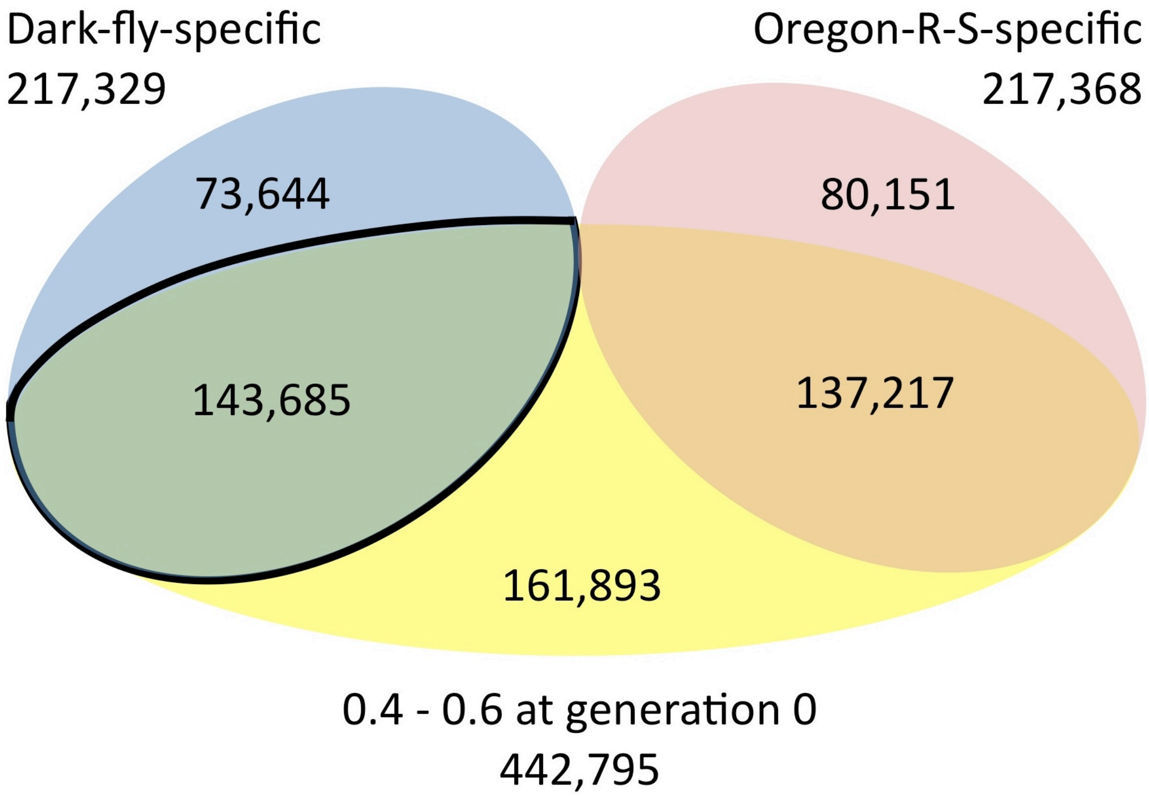

Figure S2 Comparison of SNPs identified in the mixed population genome and in the previous genome sequencing

A Venn diagram showing the relationships between SNPs showing frequency 0.4 - 0.6 in the mixed population at generation 0, and SNPs identified in Dark-fly or in Oregon-R-S in previous genome analyses (Izutsu et al., 2012). We subjected 143,685 Dark-fly's SNPs (black-outlined area) that showed frequency 0.4 to 0.6 at generation 0 to subsequent analyses. The number of strain-specific SNPs was slightly different from that shown in the previous report, due to using different filters for SNP identifications. We found that most of the SNPs (light-yellow area) that showed frequency 0.4 - 0.6 at generation 0 but that have been identified neither in Dark-fly nor Oregon-R-S were located in low-coverage regions in the previous sequencing.
