# Supplementary material for: Dynamics of Dark-Fly Genome Under Environmental Selections
Source: G3 (Bethesda). 2015 Dec 4;6(2):365–76. doi: 10.1534/g3.115.023549 (PMC4751556; doi:10.1534/g3.115.023549)
Supplement: Supporting Information [file supp_g3.115.023549_FigureS3.pdf]

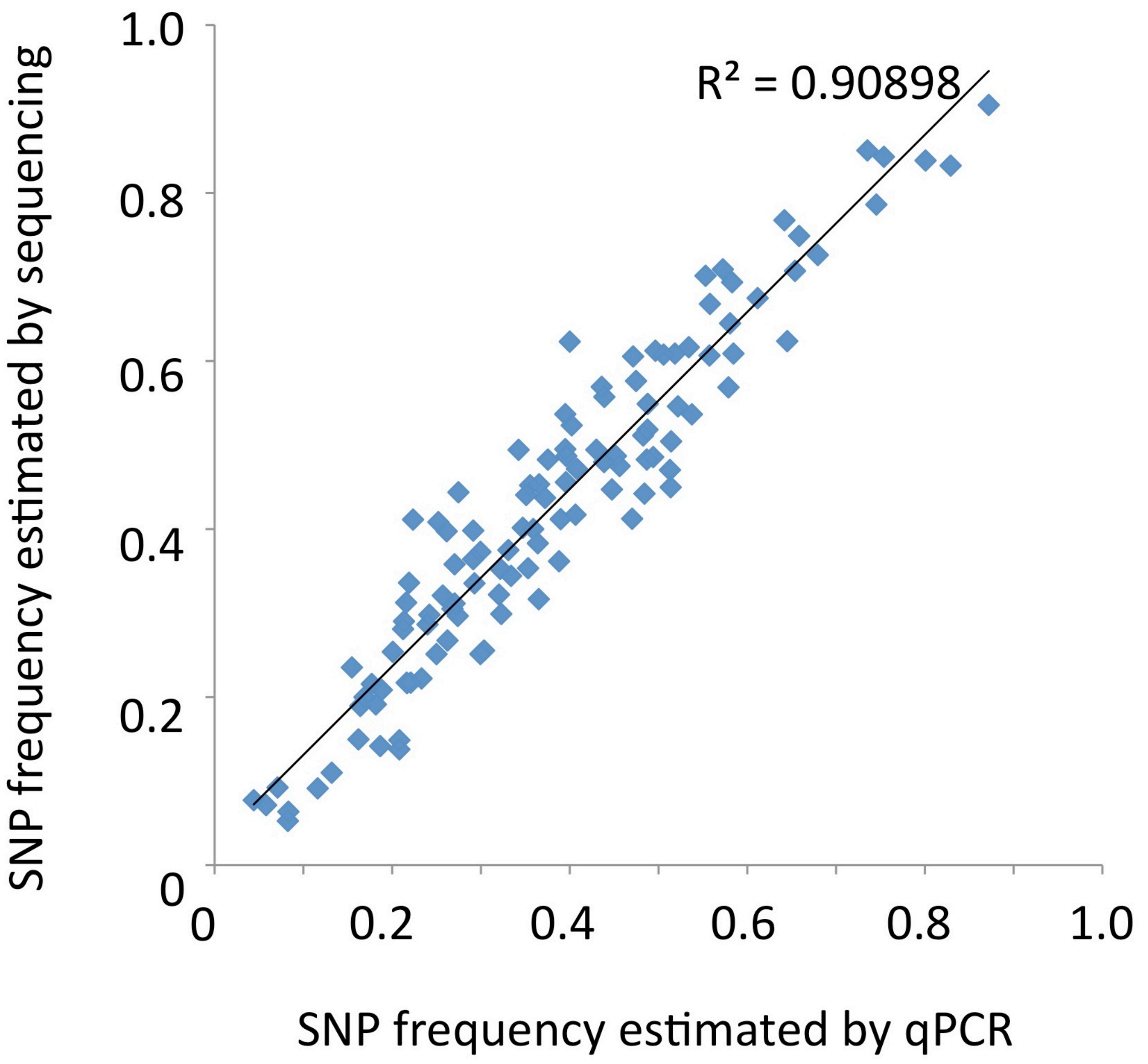

Figure S3 Reliability of SNP frequency data

Scatter plot comparing SNP frequency estimated by NGS sequencing (y-axis) and by qPCR (x-axis). Measurements from 116 samples were plotted. The estimates were strongly correlated with each other.
