# Supplementary material for: Dynamics of Dark-Fly Genome Under Environmental Selections
Source: G3 (Bethesda). 2015 Dec 4;6(2):365–76. doi: 10.1534/g3.115.023549 (PMC4751556; doi:10.1534/g3.115.023549)
Supplement: Supporting Information [file supp_g3.115.023549_FigureS4.pdf]

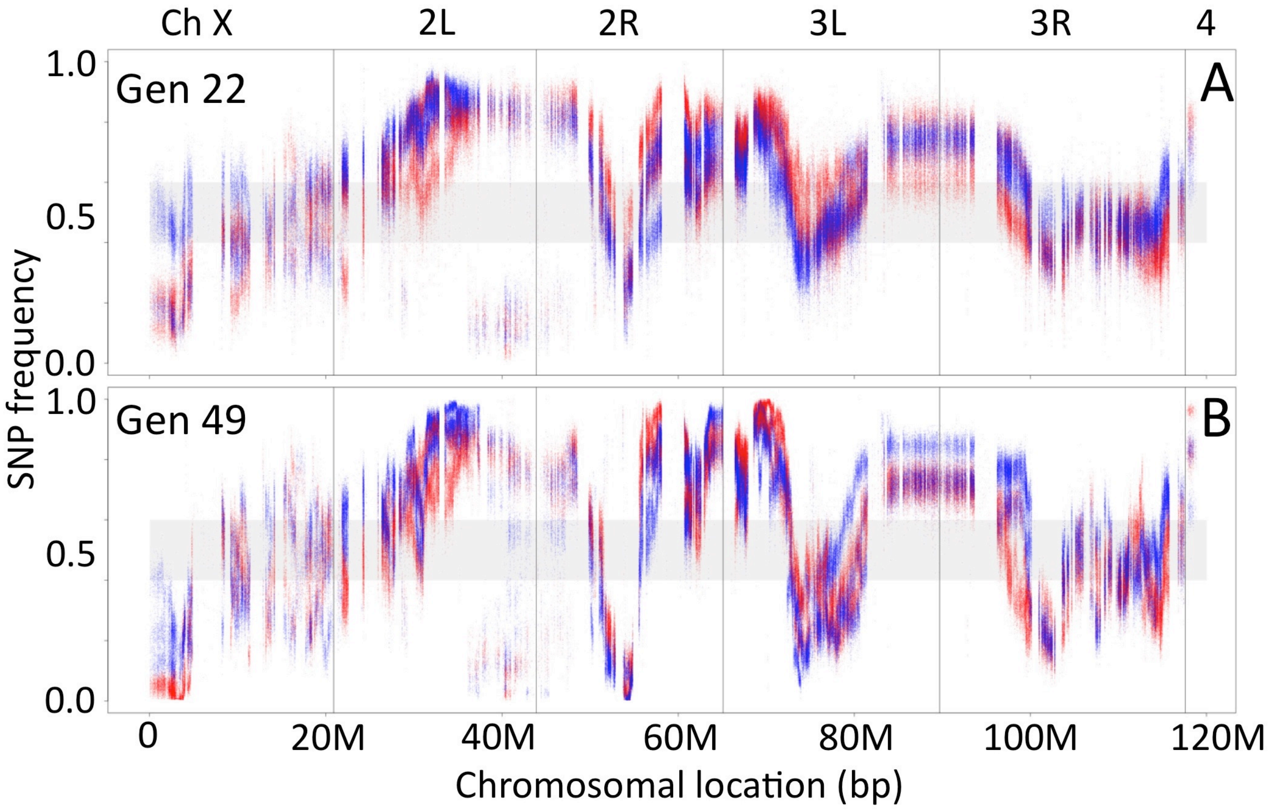

Figure S4 SNP frequency along chromosomal position

Frequencies of SNPs in each replicate population were plotted along chromosomal position at generation 22 (A) and 49 (B). X, 2L, 2R, 3L, 3R and 4 are the names of the chromosome arms of *Drosophila*. Red and blue points showed frequencies in LD and in DD, respectively. Gray bar indicates the SNP frequency at generation 0 (0.4 - 0.6).
