# Supplementary material for: Dynamics of Dark-Fly Genome Under Environmental Selections
Source: G3 (Bethesda). 2015 Dec 4;6(2):365–76. doi: 10.1534/g3.115.023549 (PMC4751556; doi:10.1534/g3.115.023549)
Supplement: Supporting Information [file supp_g3.115.023549_FigureS5.pdf]

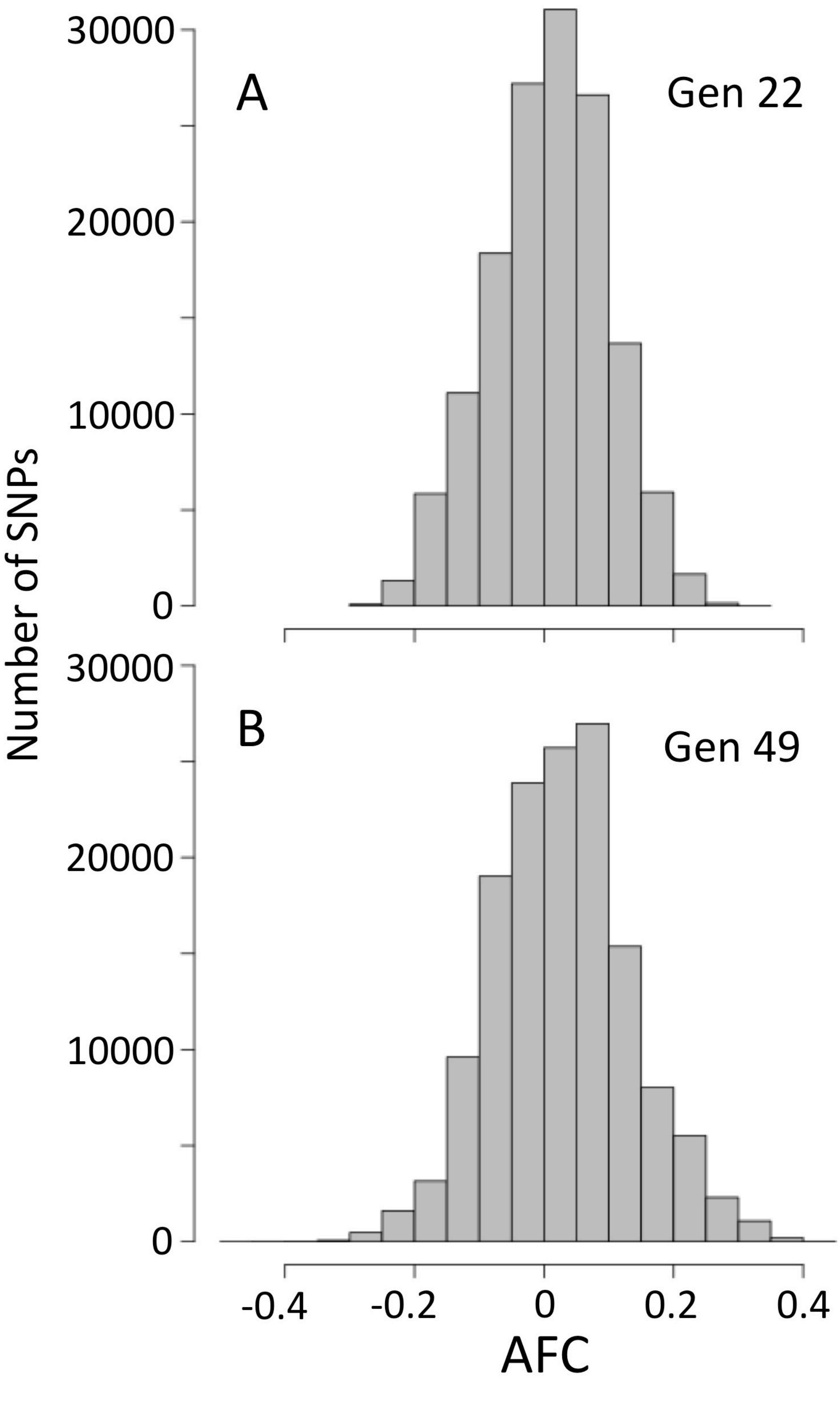

Figure S5 Histogram of allele frequency change (AFC) between LD and DD populations

Mean AFCs (frequency in DD minus frequency in LD) were 0.00802 and 0.0279 at generation 22 (A) and 49 (B), respectively.
